# State-Dependent Decoupling of a CREM-Centric cAMP-Responsive Regulon and Survival in Hepatocellular Carcinoma

**DOI:** 10.7150/ijms.140851

**Published:** 2026-10-02

**Authors:** Hao Ling, Yanzhu Hu

**Affiliations:** Department of Surgery, Klinikum rechts der Isar, TUM School of Medicine and Health, Technical University of Munich, Munich, Germany.

**Keywords:** hepatocellular carcinoma, tumor-infiltrating lymphocytes, single-cell RNA sequencing, cAMP-responsive element modulator (CREM), transcription-factor regulon, long-read sequencing

## Abstract

**Background:**

The cAMP-responsive element modulator (CREM) has emerged as a cAMP-responsive transcriptional node associated with dysfunctional lymphocyte states in chronic hepatic infection and some immunosuppressive tumor microenvironments, but its regulatory output and isoform usage by specific cell-types remain insufficiently defined in hepatocellular carcinoma (HCC). Because CREM produces activating isoforms and repressive inducible cAMP early repressor (ICER) isoforms through alternative promoter usage, bulk or single-cell CREM mRNA abundance may not faithfully describe the net direction of downstream CRE-dependent transcriptional programs.

**Methods:**

Single-cell RNA-seq datasets spanning 18,665 T cells from two independent, pathologically validated primary liver cancer cohorts (32 HCC and 5 iCCA patients) were integrated, with HCC-only sensitivity analyses confirming the primary findings. Using prior-knowledge transcriptional regulon frameworks cross-referenced with CRE-family genomic occupancy data (ENCODE), CREM mRNA expression was decoupled from its inferred downstream regulon activity at single-cell resolution. Validation was performed using a third-generation long-read direct RNA sequencing hepatic model and bulk TCGA clinical survival cohorts.

**Results:**

Across multiple regulon definitions, single-cell CREM mRNA and regulon activity were characterized by prominent low-coupling at the single-cell level (meta-Spearman ρ < 0.20, explaining <4% of variance). Crucially, this low-coupling was not uniform but followed a continuous T-cell state gradient, where the correlation between CREM expression and target activation progressively tightened as T cells transitioned from a resting state toward an activated or exhausted phenotype. Long-read direct RNA sequencing in a hepatic lineage reference model revealed that the activation-associated P1 promoter (CREMτ) predominated over the repressor-associated P2 promoter (ICER) by a 14:1 ratio under steady states. In bulk TCGA-LIHC data, elevated CREM-centric regulon scores, but not raw mRNA levels, correlated with favorable overall survival (HR = 0.787, p = 0.026), operating primarily as a T-cell activation- and infiltration-associated prognostic feature rather than an independent cell-autonomous biomarker.

**Conclusions:**

Single-cell CREM mRNA abundance is not a reliable proxy for its transcriptional output in HCC tumor-infiltrating lymphocytes. Instead, the coupling between CREM expression and its regulon activity is dynamically modulated by the T-cell activation state, a phenomenon partially explained by the usage of alternative isoforms in reference models. These findings caution against the routine practice of translating crude transcription factor mRNA metrics directly into functional regulatory inputs within a complex tumor immune microenvironment.

## Introduction

Hepatocellular carcinoma (HCC) is one of the most aggressive malignancies worldwide. HCC is characterized by high recurrence rates and limited long-term responsiveness to current immune checkpoint blockade therapies 1-3. This clinical challenge in treatment is intimately linked to the tumor immune microenvironment, which represents a highly orchestrated state of immunological tolerance and T-cell dysfunction 1, 4. Accumulating evidence from high-impact single-cell and spatial transcriptomic architectures underscores that myeloid lineage cells, mainly TREM2+ tumor-associated macrophages (TAMs) and myeloid-derived suppressor cells (MDSCs), exert a dominant role in driving CD8+ T-cell exclusion and functional exhaustion 5, 6. However, although these extrinsic myeloid-derived suppressive vectors have been extensively characterized, the precise intracellular transcriptional circuitry through which infiltrating T cells process and execute these microenvironmental stress cues remains incompletely understood.

Mechanistically, diverse extrinsic inhibitory signals delivered by the tumor milieu frequently converge on the adenosine receptor axis and pro-inflammatory prostaglandin E2 signaling cascades 4, 7. Upon ligation, these pathways activate intracellular adenylate cyclase, driving sustained elevations in cyclic adenosine monophosphate (cAMP) levels and subsequent protein kinase A activation within tumor-infiltrating lymphocytes (TILs) 8. Recent evidence has demonstrated that these components function as critical, convergent negative regulators of proximal T-cell receptor (TCR) signaling in intra-tumoral T cells. Distinct from this proximal kinase inhibition, cAMP signaling also represses effector genes directly at the promoter level, as exemplified by CREMα-mediated epigenetic remodeling and silencing of the interleukin-2 promoter, thereby paralyzing cytolytic effector programs 9. At the transcriptional terminus of this intracellular suppression cascade lies the cAMP-responsive element modulator (CREM), a terminal nuclear responder crucial for processing chronic microenvironmental cAMP stress 10. Thus, a better understanding of the downstream regulatory programs governed by CREM is essential to deciphering the underlying T-cell dysfunction in liver oncology.

Despite its strategic position at the confluence of immune suppression, the exact regulatory output of CREM in HCC lymphocytes is hindered by molecular complexity. Although recent single-cell atlases have routinely cataloged CREM transcript abundance as a standard biomarker for CD8+ T-cell exhaustion, this direct abundance-to-activity extrapolation introduces a critical limitation in conventional analytical strategies. Through alternative promoter usage, the CREM gene yields functionally distinct isoforms with opposing regulatory directions 11. Specifically, the upstream P1 promoter drives the synthesis of full-length transcriptional activators such as CREMτ. Conversely, an intronic promoter directs the expression of the inducible cAMP early repressor (ICER), a truncated, dominant-negative isoform that lacks the transactivation domain but retains DNA-binding capabilities 12, 13. Because CREM generates multiple activator and repressor isoforms through alternative promoters and splicing, and stress can induce repressor isoforms such as ICER in a time- and cell-type-dependent manner, measurements based only on total CREM mRNA abundance can miss isoform-specific regulatory directionality of downstream targets. In such settings, short-read RNA sequencing has limited ability to resolve full-length transcripts, promoter coupling, and complex isoform usage, which can bias interpretation of the underlying transcriptional landscape 14-17.

CREM and CREB1 are structurally related members of the basic leucine zipper transcription factor family and are encoded by distinct genes 18. They show strong sequence conservation, especially in regions implicated in DNA binding and dimerization 18. Both factors selectively recognize cAMP response elements (CREs), and structural work on CREB indicates that key determinants of DNA binding and family-selective dimerization are conserved across the CREB/CREM/ATF-1 family 19. This cross-talk dictates that evaluating CREM function based solely on mRNA expression frequently confounds gene-specific regulatory inputs. To circumvent this, we developed a computational constraint strategy incorporating prior-knowledge-based network topologies and shared genomic occupancy data. This approach allows us to infer a broader, CREM-centric cAMP-responsive regulon rather than an isolated single-factor signal. By systematically decoupling single-cell transcript levels from inferred network outputs across multiple pathologically validated HCC cohorts, this study provides a necessary paradigm correction for interpreting downstream cAMP-dependent transcriptional programs within the complex tumor immune microenvironment.

To address these interconnected challenges, in this study, we performed a systematic, network-constrained multi-omic exploration integrating single-cell transcriptomics, prior-knowledge-based network topologies, long-read direct RNA sequencing, and large-scale bulk clinical survival cohorts. We uncovered a prominent and pervasive low-coupling between single-cell CREM mRNA levels and actual downstream regulon activity, indicating that transcript volume is an unreliable proxy for defining functional nuclear output. Intriguingly, this low-coupling is dynamically modulated along a continuous, state-dependent T-cell activation gradient rather than being stochastically distributed. Furthermore, our long-read transcriptomic profiling corroborated a stark baseline asymmetry in alternative promoter usage, with the activating transcript vastly outnumbering the repressor transcript under steady states. Finally, applying this network-constrained framework to clinical data revealed that the CREM-centric regulon activity, rather than raw bulk mRNA, functions as a powerful T-cell activation-associated prognostic feature that effectively stratifies the overall survival (OS) of patients. Our work provides a critical methodological framework and a conceptual shift, cautioning against simplified extrapolation from transcript abundance to molecular function and delineating the internal logic of cAMP-responsive transcription in liver cancer.

## Methods

### Single-cell RNA-seq cohorts and pre-processing

Two publicly available scRNA-seq cohorts of primary liver tumors were integrated: GSE125449 20 (12 discovery-cohort samples, Set 1 of Ma et al.: 7 HCC and 5 intrahepatic cholangiocarcinoma [iCCA] patients, as defined in the original publication) and GSE151530 21 (of the 46 samples deposited under this accession, which also include iCCA biospecimens, we analyzed the 32 HCC samples from 25 patients, including multiregion biopsies).^20^, ^21^ T cells were identified by canonical T-cell marker-score annotation (CD3D/CD3E/CD3G/CD2) in GSE125449 and by the cell-type annotation of the original publication in GSE151530, yielding 1,452 and 17,213 T cells respectively (18,665 total; 44 samples from 37 patients). FOXP3 and CD56 were not used as exclusion gates; both markers entered only the downstream subtype annotation (Treg and NK-like T, respectively). Patient metadata was matched via sample barcodes with full coverage. Primary analyses used all 44 samples; sensitivity analyses restricted to HCC-only samples and with same-patient samples merged are reported in Tables S1 and S2.

### Quality control

Cells were filtered to retain those with 200-6000 detected genes, mitochondrial content <20%, and total UMI counts between the 0.5th and 99.5th percentile within each cohort. Genes detected in fewer than 3 cells were removed. Counts were log1p-normalized to 10,000 transcripts per cell using scanpy 22. The intersection of highly variable genes (HVGs, seurat_v3 method) from both cohorts was used for downstream integration, supplemented with a 29-gene bZIP/CREB pathway whitelist including CREM (full list in Table S6).

### Cohort integration

Cohorts were integrated using scVI (scvi-tools v1.1) 23 with 2 hidden layers, 30 latent dimensions, negative binomial gene likelihood, and batch key set to cohort. Models were trained with early stopping (patience = 15 on validation ELBO) at a batch size of 512. UMAP embedding was computed on the 30-dimensional 24 latent space with 15 neighbors and min_dist = 0.3. Integration quality was assessed using iLISI and kBET; Harmony integration was included as a sensitivity control. Marker-gated subtype labels (rather than cluster-derived labels) were used for all downstream analyses to mitigate residual batch structure.

### T-cell subtype assignment

Thirteen T-cell subtypes were annotated via marker-score gating on log-normalized expression: CD4 NR4A⁺ early-activated, Effector memory CD4, Effector memory CD8, CD4 other, Exhausted CD8, Naive/CM CD4, Treg, CD8 other, Cytotoxic CD8, Proliferating CD4, NK-like T, Proliferating CD8, and Naive/CM CD8. Marker panels were applied identically across both cohorts. Subtypes with ≥30 cells and ≥3 patients (each contributing ≥10 cells), with at least one qualifying patient in each cohort, were included in the formal two-cohort meta-analysis (10 of the 13 subtypes).

### Regulon construction and single-cell scoring

Three CREM regulon definitions were used:

- CollecTRI-87: Signed CREM-target relationships extracted from the CollecTRI database 25, with self-loops removed (87 final targets).
- ChIP_HepG2-27: A subset of 27 CollecTRI targets with a CREB1 IDR peak within 1 kb of the TSS in ENCODE HepG2 ChIP-seq (ENCSR000EZQ) 26.
- ChIP_either-42: A subset of 42 targets with a CREB1 peak in either HepG2 or GM12878 (ENCSR000EHM).

Per-cell regulon activity was scored using the univariate linear model (ulm) from decoupler-py with a minimum target threshold of 5. WSUM and MLM scoring methods were included as sensitivity controls.

### CREB1 ChIP-seq integration

ENCODE CREB1 IDR peaks for HepG2 and GM12878 were intersected with GENCODE v45 27 canonical TSS ± 1 kb regions of CollecTRI targets. The distance from the CREM TSS to the nearest HepG2 CREB1 peak was calculated to confirm autoregulatory locus occupancy.

A diagonal validation test was performed by comparing per-subtype meta-ρ values from CollecTRI-87 versus ChIP-anchored regulons via Pearson correlation. Empirical p-values were generated by permutation against 200 random target subsets of equal size.

### Long-read isoform classification (HepG2)

SG-NEx HepG2 direct RNA sequencing data (replicate 6) was aligned to GRCh38 using minimap2 (splice mode) 28. Sense-strand reads overlapping the CREM locus (chr10:35,121,790-35,156,790) were classified by their 5' alignment position, as follows: P1 (CREMτ activator), 5' end within ±500 bp of the canonical TSS, or P2 (ICER repressor), 5' end within +12 to +25 kb downstream of the TSS. The 95% Wilson confidence interval (CI) for the P1 isoform proportion was calculated via Newcombe's score interval. Direct cDNA data was used as a strand-bias control.

### Systematic screen of public T-cell long-read datasets

Public T-cell long-read transcriptome datasets (Oxford Nanopore direct RNA/cDNA and PacBio) were identified by systematic search of GEO, SRA, ENA and ArrayExpress. For each dataset, CREM-locus evidence (GRCh38 chr10:35,121,790-35,156,790) was classified by promoter of origin. For isoform-level datasets (TRAILS 29, Woolley et al. 2025 30, Woolley et al. 2022 31), collapsed isoforms were classified by 5'-end coordinate: P1, 5' end within CREM TSS ± 1 kb (chr10:35,125,790-35,127,790); P2/ICER, 5' end within TSS +12 kb to +25 kb (chr10:35,138,790-35,151,790); other-sense; or antisense. For read-level datasets (E-MTAB-10797 32, Fanari et al. 2025 33), reads were aligned to GRCh38 with minimap2 28 using the splice-aware preset, and locus reads carrying at least one spliced intron (CIGAR 'N') were classified by the same coordinate windows; unspliced reads were flagged as consistent with nascent transcripts. The P1 window used here (±1 kb) is wider than in the HepG2 pilot (±500 bp); across all screened T-cell datasets, only two P1-classified features have 5' ends within 500-1,000 bp of the TSS (one E-MTAB-10797 read; one Woolley et al. 2025 isoform), so promoter classifications are effectively insensitive to this window difference. Datasets with fewer than 20 spliced locus reads or isoforms were considered below the quantitative-evaluability threshold and are reported for completeness only. The full screening record, including datasets that could not be screened (data available on request; locus coverage too sparse; file corruption during transfer), is provided in Table S3.

### TCGA-LIHC bulk prognostic validation

Level-3 RSEM-normalized expression and clinical data for TCGA-LIHC primary tumors were filtered to select patients with complete age, sex, AJCC stage, and OS records (n = 330, 110 events; stage IV patients were excluded to avoid singular Cox fits).

For the anti-PD-1-treated HCC bulk RNA-seq cohort (GSE202069; 66 samples from 44 patients, of whom 17 received anti-PD-1 therapy), TPM-normalized expression was obtained from GEO, and the analysis set was restricted to the dedicated anti-PD-1 treatment series: 16 treated patients (PT-series) with both expression data and RECIST response annotation (8 responders [CR/PR], 8 non-responders [SD/PD]) curated from Supplementary Table S1 of the depositing publication (Li et al., 2024, Hepatology; ref. 50), a study that established metabolic subtypes in HCC rather than serving as the original trial report for this checkpoint cohort. One additional treated patient (ST8) belonged to the surgical cohort and was not included. A second candidate anti-PD-1 cohort, GSE285963, was considered at the pre-registered stage but excluded because all patients received combination atezolizumab-bevacizumab therapy, a non-orthogonal design that cannot isolate anti-PD-1-specific response signals.

Bulk regulon scores were computed via decoupler ulm on log1p(RSEM+1) expression. Adjusted Cox proportional-hazards models (covariates: z-scored age, sex, stage II/III indicators) were fit using lifelines. Proportional hazards assumptions were verified via Schoenfeld residuals. Survival differences across score tertiles were tested via the log-rank test. 1000-round patient bootstrap and 2-fold held-out cross-validation were performed to assess robustness.

Cell-type marker proxy scores were computed for CD8 T, NK, CD4 T, Treg, hepatocyte, endothelial, and stromal compartments to evaluate cellular confounding of the bulk survival signal.

### Statistical analysis

Per-cell Spearman correlation (ρ) between CREM mRNA (log1p-normalized) and regulon activity was computed both across all cells and restricted to CREM⁺ cells (≥1 raw UMI) to account for dropouts.

For subtype-level analysis, cohort-specific ρ values were combined via inverse-variance-weighted fixed-effect meta-analysis on Fisher-z-transformed correlations, with 95% CIs from the normal approximation. Between-cohort heterogeneity was quantified with Cochran's Q and *I²* statistics. Benjamini-Hochberg (BH) FDR correction was applied within each regulon across the 10 subtypes.

Patient-level robustness was evaluated separately per cohort via linear mixed-effects models (regulon_score ~ CREM_log1p with a random intercept per patient; maximum-likelihood fits in statsmodels), restricted to patients contributing ≥10 cells and applied to subtypes with ≥3 such patients and ≥30 eligible cells (otherwise pooled fixed-effect OLS). Because GSE151530 includes multiregion biopsies, sample-level patient identifiers were used in the primary analysis; a sensitivity analysis with same-patient samples merged (25 patients) yielded concordant estimates (Table S2).

## Results

### Cell-state composition and CREM expression across two HCC T-cell cohorts

We integrated two public primary liver cancer scRNA-seq cohorts (GSE125449, 12 samples from 12 patients: 7 HCC and 5 iCCA; GSE151530, 32 HCC samples from 25 patients) into a unified atlas of 18,665 tumor-infiltrating T cells (44 samples from 37 patients), integrated via scVI. Thirteen canonical T-cell subtypes were annotated by marker-score gating independent of embedding geometry, and projected onto the scVI UMAP layout (Figure 1A). CREM transcript expression was distributed across the T-cell manifold (Figure 1B), with elevated detection rates and expression levels in Treg, exhausted CD8, and proliferating subsets (Figure 1C, D).

Cohort and patient-level structure was validated as follows: the two cohorts shared a single continuous latent manifold rather than forming separate clusters (Figure 2A-C); CREM detection rates varied by patient and subtype (Figure 2D); and CREM expression distributions by subtype were consistent across cohorts (Figure 2E), with no systematic patient-level batch bias (Figure 2F).

Full integration quality control metrics (iLISI, kBET, silhouette score) and subtype annotation QC are provided in Figures S1 and S2.

### CREM mRNA was decoupled from CREM-regulon activity in pooled cells

We scored per-cell CREM regulatory activity using three independent regulon definitions: the literature-curated CollecTRI-87 regulon and two subsets anchored to ENCODE CREB1 ChIP-seq peaks (ChIP_HepG2-27, ChIP_either-42). Regulon composition details are summarized in Figure S3A.

Across all 18,665 cells, Spearman's correlations between CREM mRNA and regulon activity were consistently weak for all three regulons, as shown by hexbin density plots: ρ = 0.117 for CollecTRI-87 (Figure 3A), 0.164 for ChIP_either (Figure 3D), and 0.095 for ChIP_HepG2 (Figure 3E), with mRNA explaining less than 4% of variance in regulon activity. Correlation values were concordant between the two independent cohorts (Figure 3B, C), and remained low when restricted to CREM⁺ cells (≥1 raw UMI) to control for dropout (Figure 3F). The same dissociation pattern was independently replicated in bulk TCGA-LIHC data (Spearman ρ = 0.022, p = 0.7; Figure 8E), confirming the finding was not an artifact of single-cell dropout.

Sensitivity analysis using alternative scoring methods (WSUM, MLM) yielded concordant results.

### Cell-state-specific dissociation with rest-to-activation gradient

For 10 subtypes meeting the minimum cell-count and patient-diversity thresholds, we computed cohort-meta Spearman ρ via inverse-variance-weighted fixed-effect meta-analysis with BH-FDR correction. Full per-subtype statistics are provided in Table 1.

Coupling strength formed an ordered gradient aligned with T-cell activation state. Forest plots of meta-ρ ranked from lowest to highest showed near-zero coupling in Proliferating CD4 (meta-ρ = -0.009), increasing gradually through Naive/CM CD4, Effector memory CD4, and Treg, and peaking in CD4 NR4A⁺ early-activated and Effector memory CD8 subsets (all ρ < 0.2; Figure 4A). The same gradient was reproduced using the ChIP_either-42 regulon (Figure 4B). Dissociation index (1 - |ρ|) values mirrored this rest-to-activation trend (Figure 4C). Between-cohort heterogeneity was low for most subtypes (median I² = 0%; Figure 4D), and per-subtype ρ values were concordant across cohorts (Figure 4E). Subtype cell counts and statistical significance are summarized in Figure 4F-G. Of 30 subtype × regulon tests, 23 reached BH-FDR q < 0.05.

Patient-level robustness was confirmed by per-sample × per-subtype correlation heatmaps (Figure 7A) and per-subtype distributions of per-sample ρ values (Figure 7B), which recapitulated the same activation-state gradient. Linear mixed-effects models with patient as a random intercept further validated the gradient was not driven by individual patients (Figure 7C), with consistent counts of FDR-significant subtypes across all three regulons (Figure 7D).

Extended per-cohort statistics, bootstrap CIs, and full mixed-effects model outputs are provided in Table S4.

### CREB1 ChIP anchoring supports the specificity of the dissociation pattern

To exclude literature-curation bias, CollecTRI-87 targets were intersected with ENCODE CREB1 ChIP-seq peaks from HepG2 and GM12878 cell lines. Genome browser tracks confirmed CREB1 peak occupancy at the CREM promoter and three representative target loci (Figure 5A1-A4). The closest HepG2 CREB1 peak to the CREM transcription start site was only 92 bp away, indicating CRE-family transcription factors could directly bind the CREM promoter region (Figure 5D).

Per-subtype meta-ρ values from ChIP-anchored regulons were highly concordant with those from CollecTRI-87, shown by diagonal-test scatter plots: Pearson r = 0.882 for ChIP_HepG2-27 and r = 0.959 for ChIP_either-42 (Figure 5B). Permutation testing against 200 random target subsets confirmed this concordance was far above background for ChIP_either-42 (empirical p < 0.005; Figure 5C). The three regulon sets formed a fully nested structure (Figure 5E), confirming the dissociation pattern was robust to target trimming and concentrated on directly chromatin-bound targets.

Full lists of ChIP-anchored target genes are provided in Table S5.

### Long-read sequencing shows activator-isoform dominance in HepG2 cells

As no public long-read T-cell datasets with sufficient CREM-locus coverage were available at the time of the primary analysis, we used SG-NEx HepG2 direct RNA sequencing as a hepatocyte-lineage reference to assess CREM isoform usage. A systematic screen of public T-cell long-read datasets identified subsequently is presented as item (5) under 'Orthogonal sensitivity validations' below.

Per-read 5'-anchor position histograms across the CREM locus showed two distinct peaks corresponding to the P1 (CREMτ activator) and P2 (ICER repressor) promoters (Figure 6A). Of classifiable sense-strand reads, 42 mapped to P1 and 3 to P2, giving a 14:1 activator-to-repressor ratio, with P1 accounting for 93% of isoforms (95% Wilson CI [82%, 98%]; Figure 6B, F). P1 and P2 reads showed comparable read lengths and intron counts, arguing against technical fragmentation artifacts (Figure 6C-D). Strand-bias diagnostics confirmed direct RNA data provided reliable strand-specific quantification, in contrast to direct cDNA controls (Figure 6E).

Additional per-read details and direct cDNA control analyses are provided in Figure S4. This result indicates a directional constraint: in a hepatic lineage context, CREM transcripts are overwhelmingly activator-derived, and the observed mRNA-activity decoupling is unlikely to be explained by dominant repressor-isoform expression.

### CREM regulon activity, not mRNA, was associated with survival in TCGA-LIHC

We tested prognostic association in 330 TCGA-LIHC primary tumors with complete clinical follow-up. Kaplan-Meier curves stratified by regulon-score tertiles showed significantly longer OS in the high-score group (log-rank χ² = 6.79, p = 0.034; Figure 8A), whereas CREM mRNA tertiles showed no survival difference (log-rank χ² = 0.86, p = 0.65; Figure 8B).

Adjusted Cox proportional-hazards models (covariates: age, sex, AJCC stage) confirmed that higher CREM regulon score was significantly associated with longer OS. Full model statistics are provided in Table 2: regulon score HR = 0.787 per SD, 95% CI [0.637, 0.972], p = 0.026; CREM mRNA HR = 0.995, p = 0.96 (Figure 8C). Bootstrap resampling of 1000 patient replicates supported the robustness of the regulon effect (median HR = 0.786, 95% CI [0.622, 0.999]; Figure 8D). Held-out 2-fold cross-validation showed directional consistency, though effect magnitude was attenuated in one fold (Figure 8C). We observed negligible correlation between bulk CREM mRNA levels and the CREM regulon score, indicating decoupling of gene expression from downstream regulon activity (Figure 8E).

Cell-type marker proxy analysis showed regulon score correlated positively with NK and CD8 T-cell markers and negatively with stromal markers, indicating that the bulk regulon score acts primarily as a T-cell activation- and infiltration-associated feature rather than an independent cell-autonomous biomarker (Figure 8F).

### Orthogonal sensitivity validations

Six orthogonal sensitivity analyses are presented in full in the Supplementary Material (Figures S3-S4 and complete statistics in Tables S1-S3 and S6-S9), with core findings summarized as follows: (1) SCENIC de-novo regulon inference: Directionally consistent with the CollecTRI-based results but limited by bZIP-family motif degeneracy. Full per-seed regulon targets and cross-subtype comparison statistics are shown in Table S6; (2) TCR clonal expansion analysis (GSE98638, Figure 9): CREM regulon activity, not mRNA, was positively associated with clone size in Effector memory CD4 T cells, consistent with the activation-state gradient. Full per-subtype statistics and mixed-effects model outputs are provided in Table S7; (3) T-cell long-read pilot (TRAILS 29 DRR465281): Pre-specified read-depth threshold was not met; no definitive T-cell isoform conclusion could be drawn. Read classification counts are shown in Table S8; (4) Anti-PD-1 bulk cohort analysis (GSE202069, Figure 10): No significant association was observed between bulk regulon score and immunotherapy response, likely due to tissue dilution of the T-cell-specific signal at this sample size. Full per-patient statistics are in Table S9; (5) T-cell promoter usage at the CREM locus in public long-read datasets: To test whether the P1-dominant promoter usage observed in HepG2 is cell-line-specific, we systematically screened all publicly available T-cell long-read transcriptome datasets (Methods; Table S3). Five datasets yielded classifiable CREM-locus evidence. In TRAILS (15 T-cell subsets plus PBMC), all 22 CREM-locus transcripts initiate at P1, with P1 isoforms detected in every subset (8-61 CREM-locus reads per subset) and zero P2/ICER isoforms in the 210,019-isoform matrix. In Woolley et al. 2025 30, all 34 collapsed CREM isoforms across four activation time points initiate at P1; in Woolley et al. 2022 31, all 3 isoforms (CD4 and CD8) initiate at P1. Read-level classification of E-MTAB-10797 32 yielded 4 P1 versus 1 P2 among 17 splice-filtered locus reads (the remaining 12 reads comprising 7 other-sense and 5 antisense reads), and a partial read-level screen of Fanari et al. 2025 33 (activated T cells) yielded 48 P1 versus 0 mature P2/ICER among 50 locus reads (the single P2-window read was unspliced and consistent with a nascent transcript). Thus, across donors, T-cell subsets and activation states, CREM transcription in T cells is overwhelmingly P1-derived, mirroring HepG2 (93% P1; 95% Wilson CI [82%, 98%]) and indicating that P1 dominance is a general feature of the locus rather than a HepG2 artifact. Full per-dataset classification counts, including datasets that could not be screened, are provided in Table S3. The P1 classification window used for this screen (CREM TSS ±1 kb) is slightly wider than that used in the HepG2 pilot (±500 bp); across all screened datasets, only two P1-classified features have 5' ends within 500-1,000 bp of the TSS, so promoter classifications are effectively insensitive to this window difference (Methods); and (6) Cohort-composition and patient-clustering sensitivity: restricting GSE125449 to HCC patients only (excluding the 5 iCCA patients and their 897 T cells) changed pooled ρ by ≤0.005 and per-subtype meta-analytic ρ by at most 0.025 (22/30 versus 23/30 tests at q < 0.05; Table S1); merging same-patient multiregion samples in GSE151530 (32 samples → 25 patients) yielded generally concordant mixed-effects model estimates (Table S2).

## Discussion

Although current paradigms in liver oncology heavily focus on extrinsic myeloid-derived suppressive vectors, predominantly TREM2+ TAMs and MDSCs that drive T-cell paralysis via the hyperactivation of the intracellular cAMP/PKA axis 5, 34, 35, the precise directional downstream transcriptional wiring executed by these infiltrating lymphocytes remains a critical black box 36, 37. The potential decoupling between transcription factor transcript abundance and actual regulatory output represents a critical yet frequently overlooked blind spot in single-cell cancer immunology 25, 38. In this study, through a systematic integration of 18,665 tumor-infiltrating T cells across multiple independent HCC cohorts, we demonstrated that single-cell CREM mRNA expression is characterized by a prominent low-coupling with its downstream regulon activity (meta-Spearman ρ < 0.20). Crucially, this phenomenon appears to be modulated by a state-dependent continuous gradient, where the correlation between CREM expression and target activation progressively tightens as T cells transition from a resting state toward an activated or exhausted phenotype. By integrating prior-knowledge network constraints, shared cis-regulatory genomic occupancies, third-generation long-read direct RNA sequencing, and clinical survival cohorts, our findings challenge the conventional dogma that directly equates crude elevation of mRNA transcription with functional immune suppression, thereby providing a more nuanced perspective on intracellular transcriptional rewiring within the HCC microenvironment 25, 39.

To contextualize these findings within the macro-environmental architecture of HCC, it is essential to trace how extrinsic immunosuppressive vectors converge onto intracellular cascades. The HCC microenvironment is heavily dominated by myeloid lineage cells, particularly TREM2+ TAMs and MDSCs, which actively paralyze CD8+ T-cell cytolytic programs and drive resistance to immune checkpoint blockade 5, 40, 41. These myeloid-derived suppressive signals, alongside tumor-secreted factors, prominently trigger the adenosine-A2A/A2B receptor axis and the PGE2-EP2/EP4 signaling cascades on infiltrating lymphocytes 35, 42. This Gs-protein-coupled activation enforces a sustained elevation of intracellular cAMP and chronic activation of protein kinase A (PKA), positioning the cAMP/PKA axis as a critical, convergent intracellular bottleneck that silences distal TCR signaling 43, 44. As a terminal nuclear responder to cAMP kinetics, CREM is highly sensitive to this microenvironmental stress 10, 12, 45.

However, our third-generation long-read direct RNA sequencing analysis based on a well-characterized hepatic model revealed a stark asymmetry in promoter engagement under baseline conditions, with the activation-associated P1 promoter (driving full-length CREMτ) predominating over the repressor-associated P2 promoter (driving truncated ICER) by a 14:1 ratio. In classical molecular immunology, acute TCR stimulation triggers a rapid, transient negative feedback loop dominated by inducible ICER expression to prevent hyperactivation 12. In contrast, our transcriptomic data suggests that within the chronic, adenosine- and PGE2-rich stress environment, the cellular transcriptional machinery might undergo distinct long-term adaptations. Although these long-read data stem from an epithelial reference and warrant caution before cross-lineage extrapolation to primary immune cells, they provide a necessary proof-of-principle indication that the observed single-cell decoupling is unlikely to be solely driven by a dominant repressor isoform under steady states. Instead of executing traditional ICER-mediated active repression, the elevated CREM mRNA observed in single-cell datasets may reflect inert, compensatory transcriptional pooling elicited by chronic microenvironmental cAMP stress, explaining the low-coupling between its raw transcript levels and functional repressor outputs 45, 46.

This state-dependent low-coupling further highlights the clinically relevant prognostic signals observed in bulk patient cohorts. In our survival analysis of the TCGA-LIHC cohort, elevated CREM-centric regulon scores, rather than total CREM mRNA expression, predicted significantly favorable OS (HR = 0.787, p = 0.026). Given that this calculated regulon score exhibited a recognized positive correlation with CD8+ and NK cell infiltration densities (NK: ρ = 0.35, p = 5.2 × 10⁻¹¹; CD8⁺ T: ρ = 0.30, p = 2.7 × 10⁻⁸) and a negative alignment with stromal signatures, this prognostic asset understandably intersected with the total density of the TIL compartment. Clinically, stratified analytical perspectives, such as segregating patient cohorts based on baseline T-cell infiltration levels, indicate that the CREM-centric regulon score operates effectively as a T-cell activation-associated prognostic feature rather than an isolated, independent biomarker. In HCC and chronic liver injury settings, interpretation of bulk CREM expression is potentially confounded by non-immune parenchymal hepatocytes, because hepatocytes strongly induce CREM and CRE-dependent transcriptional responses during regeneration and inflammatory stress 47-49. This microenvironmental noise explains why raw bulk CREM mRNA levels completely fail to offer prognostic stratifications. In stark contrast, by utilizing decoupled univariate linear modeling constrained by prior-knowledge topologies and shared cis-regulatory occupancies, our regulon framework successfully de-convoluted this complex mixture, selectively extracting the functional, activation-linked transcriptional output of the intra-tumoral T-cell compartment. This key distinction further clarifies the divergence observed in advanced, late-stage anti-PD-1 therapeutic cohorts (GSE202069, deposited by Li et al. 50 within their HCC metabolic-subtype study rather than a dedicated checkpoint-blockade trial report), where the overriding signals of treatment-induced tissue remodeling, parenchymal destruction, and desmoplastic stromal fibrosis inevitably dominate bulk transcriptomic profiles, thereby masking the delicate survival signals captured by homeostatic immune regulon frameworks.

Despite the methodological and conceptual insights provided by this study, several critical core boundaries must be explicitly acknowledged. First, due to technical dropouts and the low baseline abundance of CREM in primary single-cell suspension inputs, our mechanistic anchoring leveraged ENCODE ChIP-seq genomic occupancy datasets derived from CREB1. Structurally, CREM and CREB1 are evolutionary counterparts belonging to the basic leucine zipper (bZIP) transcription factor superfamily; they share near-identical DNA-binding domains and selectively recognize the exact same symmetrical octanucleotide cAMP response element (CRE motif: 5'-TGACGTCA-3'), demonstrating extensive genomic redundancy and overlapping chromatin occupancy. Consequently, the computationally derived metrics in this study cannot completely isolate CREM-specific signals from broader CREB1/family regulatory interference, meaning the results are more accurately interpreted as a broader, CREM-centric cAMP-responsive regulon. Two further limitations of this proxy deserve explicit acknowledgment. (i) CREM is unique among the family in generating both activator (CREMτ) and repressor (ICER; α/β/γ) isoforms from alternative promoters, and CREB1 ChIP-seq cannot distinguish which paralog - or which CREM isoform - occupies a given CRE; occupancy therefore does not by itself establish activating versus repressive output. (ii) Family members differ in co-factor recruitment (CBP/p300 KIX-domain binding; CRTC/TORC interaction) and in dimerization-dependent DNA affinity, so occupancy strength at a given CRE need not be identical across paralogs. In particular, CREMτ shares with CREB1 the KID-domain phosphoacceptor that recruits CBP/p300 through the KIX domain and likewise engages CRTC/TORC coactivators through its bZIP domain, but differences in glutamine-rich-domain composition and dimerization specificity make its transactivation output at a shared CRE quantitatively paralog-dependent rather than equivalent to CREB1 48. Moreover, ICER isoforms lack these activation domains entirely and repress transcription purely through competitive occupancy of the same CRE. CREB1-anchored peaks therefore cannot distinguish activating CREMτ occupancy from repressive ICER occupancy at any given site. The net effect on our analysis is that CREB1-anchored target selection may overestimate activating occupancy at some sites and may miss CREM-unique sites; because our decoupling conclusion rests on expression-level regulon behavior rather than on occupancy alone, this bias is unlikely to account for it. Paralog- and isoform-resolved CREM ChIP-seq or CUT&RUN in T cells, ideally under controlled cAMP manipulation, will be required to settle occupancy directly. Second, our isoform-level resolution (P1 vs P2 promoter usage) was validated using an epithelial lineage cell model (HepG2) rather than primary human patient-derived TILs. Because transcription factor alternative splicing and promoter selection exhibit profound cell-lineage and stimulus-specific dynamics, we assessed how far this limitation extends by systematically screening all publicly available T-cell long-read transcriptome datasets (Methods; Table S3). Across the five datasets with classifiable CREM-locus evidence - TRAILS (15 T-cell subsets plus PBMC), Woolley et al. 2025 (four activation time points), Woolley et al. 2022 (CD4 and CD8), E-MTAB-10797, and a partial read-level screen of Fanari et al. 2025 - CREM transcription in T cells is overwhelmingly P1-derived: 22/22, 34/34 and 3/3 isoforms initiate at P1 in the three isoform-level datasets, and read-level classification found 4 P1 versus 1 P2 (E-MTAB-10797; below the ≥20-read threshold and reported for completeness only) and 48 P1 versus 0 mature P2/ICER (Fanari activated T cells; partial screen). Mature, spliced P2/ICER transcripts were not detected in any dataset at current sequencing depths. P1 dominance is therefore not a HepG2 peculiarity but a general feature of the locus across T-cell subsets, donors and activation states. Importantly, every screened dataset profiles T cells under steady-state or short-term *in vitro* activation conditions; none captures primary tumor-infiltrating T cells exposed to the chronic, *in vivo* cAMP/adenosine stress gradients of the immunosuppressive tumor microenvironment. The P1 dominance we report therefore strictly describes baseline promoter usage, and dynamic isoform shifts toward P2/ICER under tumor-microenvironmental stress remain possible and untested. Moreover, because cAMP-PKA signaling induces the repressor-encoding P2/ICER promoter, the chronically elevated cAMP of the tumor microenvironment would be expected to increase, not decrease, repressor dosage in T cells; the 93% P1 usage observed in HepG2 is thus best interpreted as an upper bound on activator-isoform dominance, and the state-dependent decoupling we report is conservative with respect to this limitation. We nonetheless acknowledge two residual caveats: no public long-read dataset profiles T cells under a controlled cAMP gradient, and current sequencing depths cannot exclude low-abundance ICER transcripts below the detection threshold. This lack of verification in human primary T cells therefore remains a substantial limitation that prevents direct causal conclusions regarding primary lymphocyte biology, and direct long-read profiling of cAMP-manipulated T cells is a priority for future work. Finally, the single-cell cohorts have compositional caveats: GSE125449 included five iCCA patients (897 of 1,452 T cells), and GSE151530 included multiregion biopsies (32 samples from 25 patients). HCC-only re-analysis and patient-merged mixed-effects models both yielded concordant results (Tables S1 and S2), indicating that the state-dependent decoupling pattern is driven by neither cohort composition nor sample-level clustering.

Nevertheless, by demonstrating a pervasive low-coupling between single-cell transcription factor expression and actual regulon outputs, this study provides a crucial paradigm adjustment for translational oncology. The findings strongly caution against the routine but flawed practice of translating crude mRNA metrics directly into functional regulatory signatures within a complex, highly suppressed tumor immune microenvironment.

## Supplementary Material

Supplementary figures and tables.

## Figures and Tables

**Figure 1:**
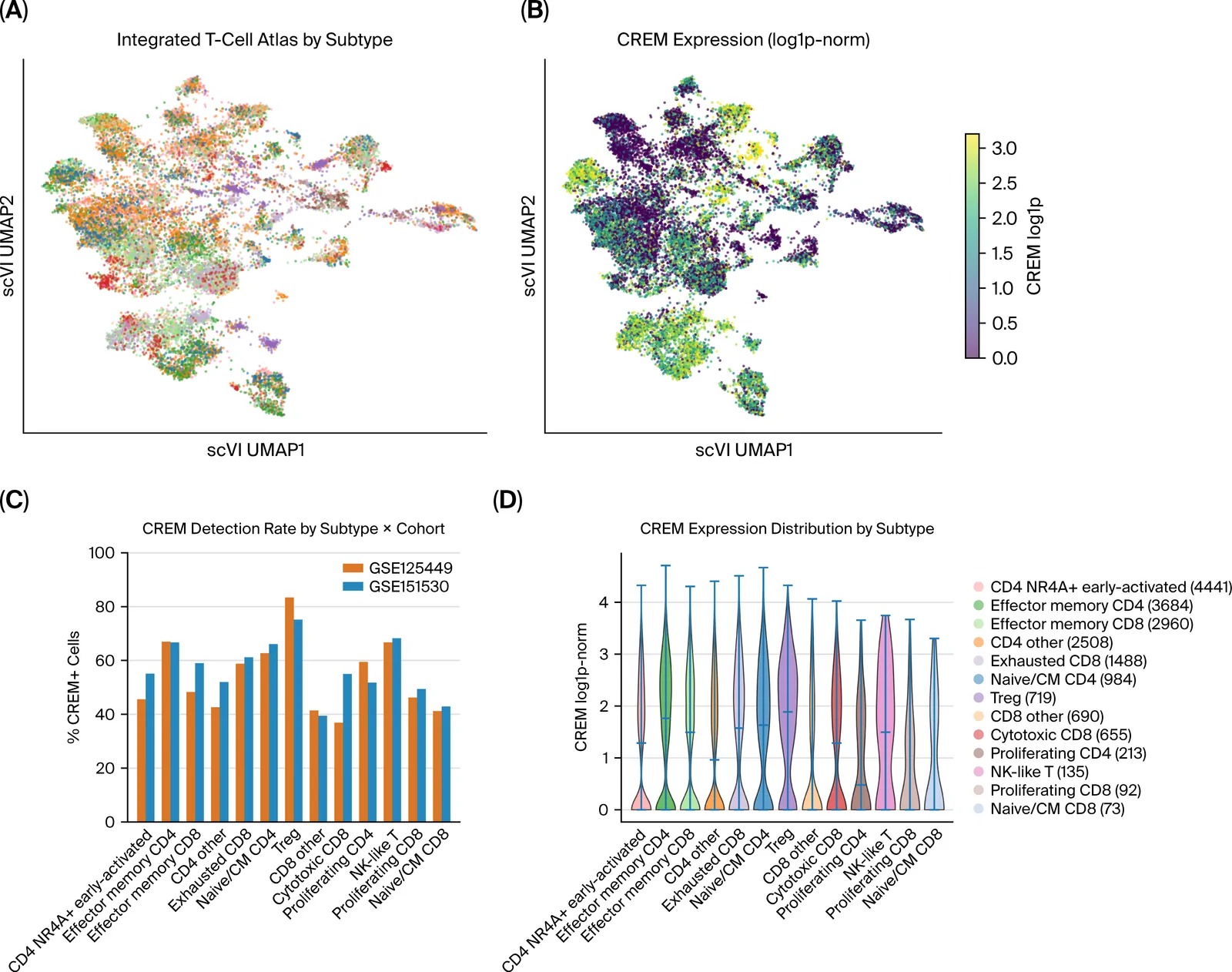
** Landscape of tumor-infiltrating T cells and CREM expression in integrated HCC cohorts.** (A) UMAP visualization of 18,665 T cells from 44 samples (37 patients; GSE125449 + GSE151530 datasets) integrated by scVI, colored by T-cell subtype, and annotated via marker-score gating (23 cells not meeting any marker threshold are labeled Unclassified). (B) Same UMAP colored by log1p-normalized CREM expression. (C) Bar plot showing the percentage of CREM⁺ cells (≥1 raw unique molecular identifier (UMI)) per subtype, ordered by cell abundance. (D) Violin plot of CREM expression distribution across subtypes. Dataset source (panels A-D): single-cell RNA-seq of HCC tumor/adjacent-liver T cells, GSE125449 (12 samples, 12 patients: 7 HCC, 5 iCCA) and GSE151530 (32 samples, 25 patients), integrated by scVI.

**Figure 2:**
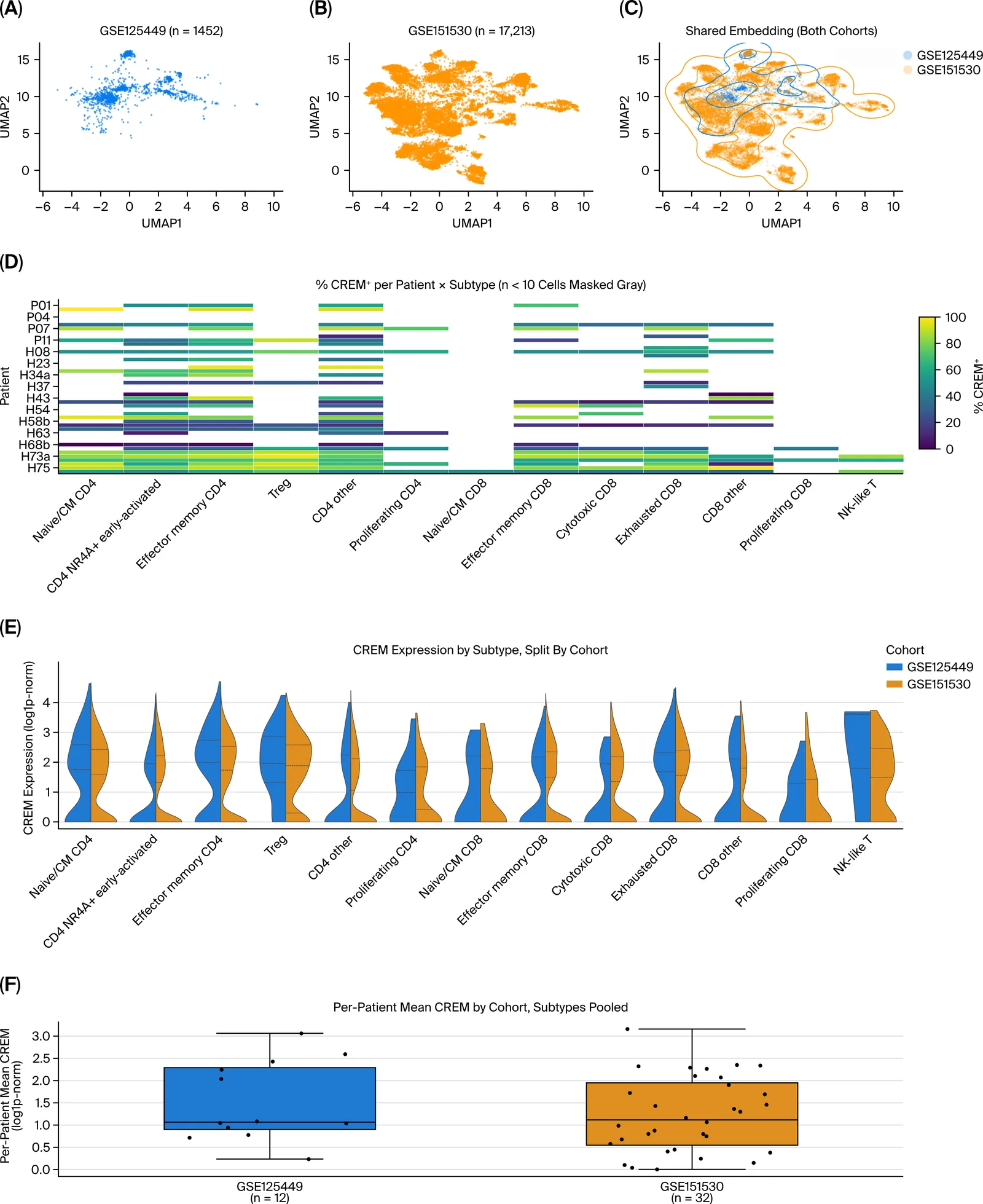
** Cohort and patient structure of the integrated T-cell atlas.** (A-B) scVI UMAP projection of T cells from the GSE125449 (A, n = 1,452 cells, 12 patients: 7 HCC, 5 iCCA) and GSE151530 (B, n = 17,213 cells, 32 samples from 25 patients) datasets separately. (C) Overlay of both cohorts on the shared UMAP embedding, with kernel density contours. (D) Heatmap showing the percentage of CREM⁺ cells per patient per subtype; entries with <10 cells are masked in gray. (E) Split violin plot of CREM expression by subtype, stratified by cohort. (F) Box-and-strip plot of per-patient mean CREM expression across cohorts, with all T-cell subtypes pooled. Dataset source (panels A-F): single-cell RNA-seq, GSE125449 (panel A) and GSE151530 (panel B); panels C-F use the integrated GSE125449 + GSE151530 atlas.

**Figure 3:**
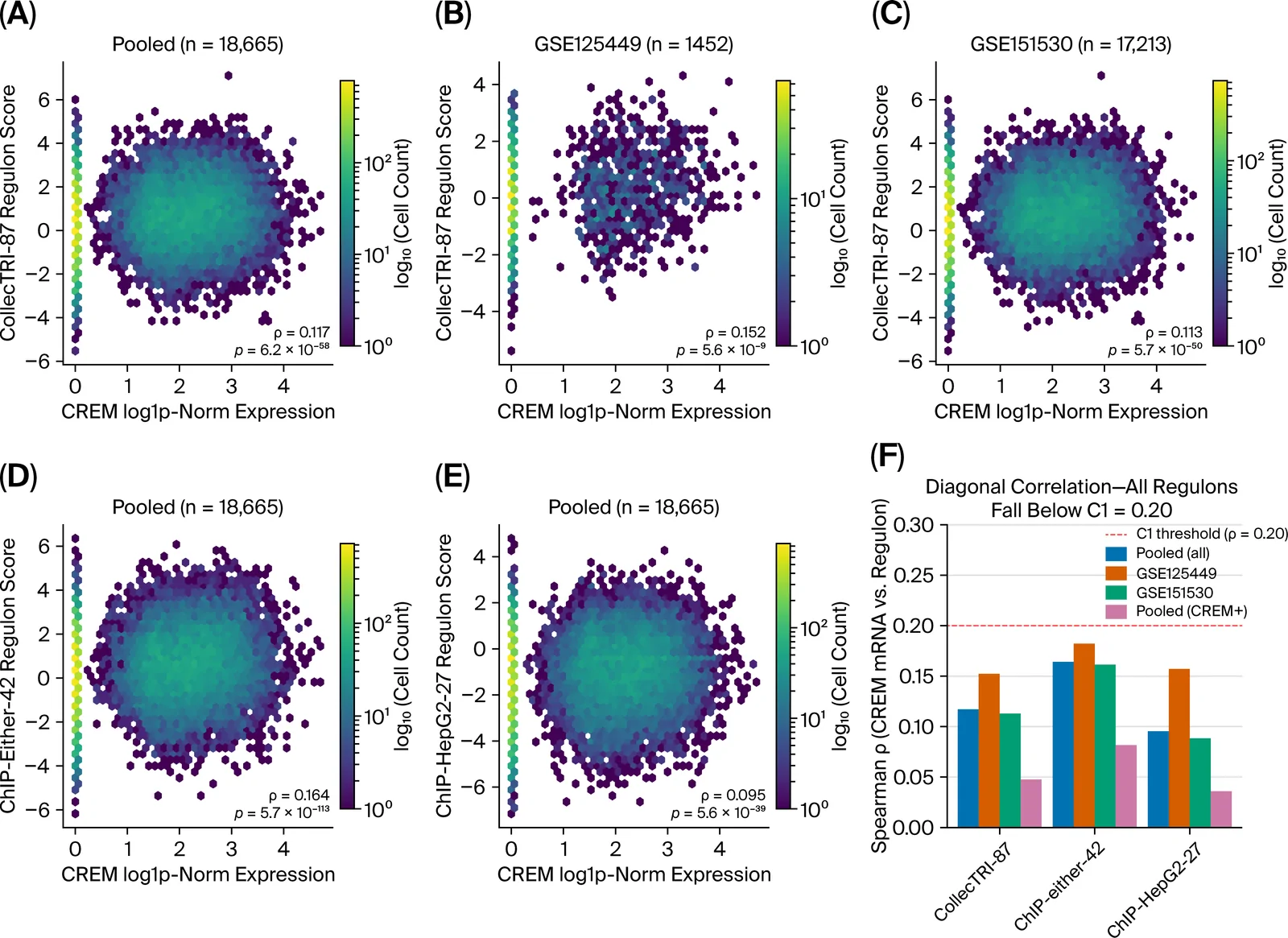
** Decoupling of CREM mRNA and CREM-regulon activity in pooled T cells.** (A-C) Hexbin density plots of CREM log1p expression versus CollecTRI-87 regulon activity score in all pooled cells (A; n = 18,665) and in each cohort separately (B: GSE125449, n = 1,452; C: GSE151530, n = 17,213). Spearman's correlation coefficients are indicated. (D-E) Hexbin density plots of CREM log1p expression versus ChIP_either-42 (D) and ChIP_HepG2-27 (E) regulon activity scores, respectively, in all pooled cells (n = 18,665). (F) Bar plot of Spearman ρ between CREM mRNA and each of the three regulon scores, computed on all pooled cells, on each cohort separately, and on CREM⁺ cells only (≥1 raw unique molecular identifier (UMI)); all values fall below the pre-specified diagonal-correlation threshold (ρ = 0.20, dashed line). Dataset source (panels A-F): single-cell RNA-seq (GSE125449 + GSE151530).

**Figure 4:**
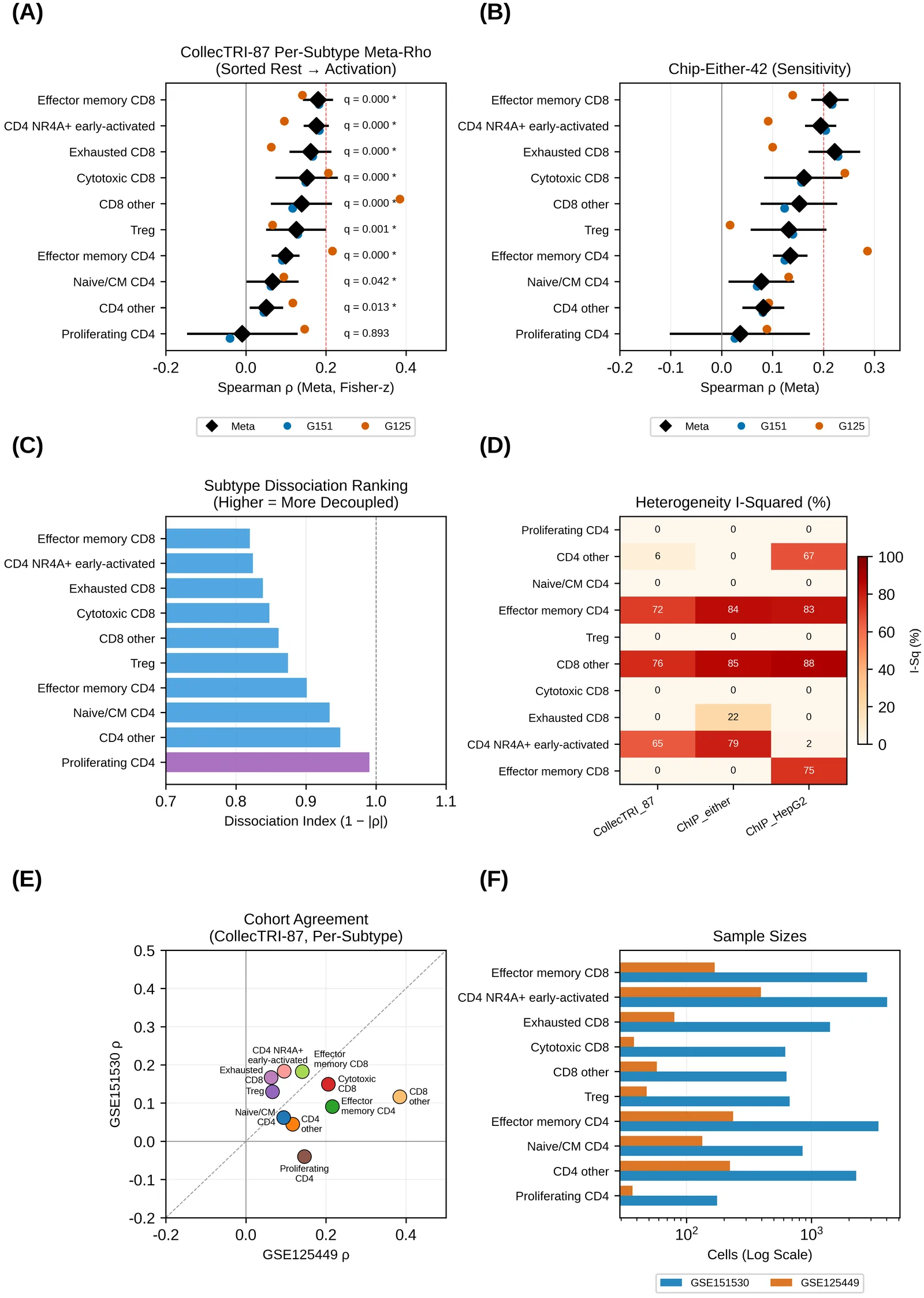
** Cell-state-specific dissociation follows a rest-to-activation gradient.** (A-B) Forest plots of per-subtype meta-analytic Spearman ρ for CollecTRI-87 (A) and ChIP_either-42 (B) regulons, ordered by ascending coupling strength. Error bars represent 95% confidence intervals (CIs) from fixed-effect inverse-variance meta-analysis. (C) Bar plot of dissociation index (1 - |meta-ρ|) per subtype. (D) Heatmap of between-cohort heterogeneity (I² statistics) across subtype × regulon tests. (E) Scatter plot comparing per-subtype Spearman ρ values between the two cohorts. (F) Cell counts for each subtype × cohort subgroup. (G) Summary annotation of statistical significance. Dataset source (panels A-G): single-cell RNA-seq, GSE125449 + GSE151530.

**Figure 5:**
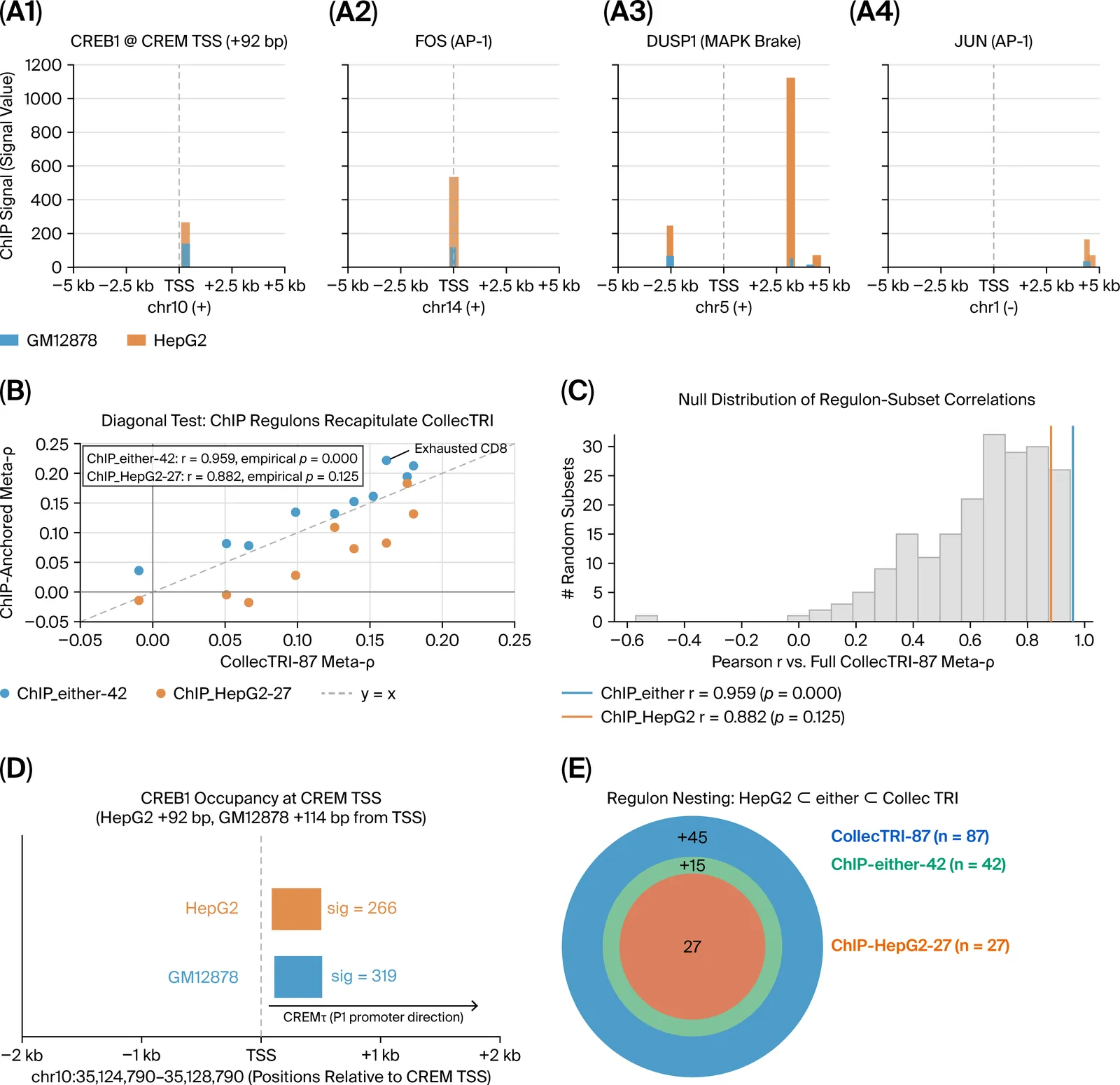
** CREB1 ChIP-seq anchoring of CREM regulon targets.** (A1-A4) Genome browser tracks of ENCODE CREB1 ChIP-seq peaks at the CREM locus (A1) and three representative target genes (A2-A4) in HepG2 and GM12878 cell lines. (B) Scatter plot comparing per-subtype meta-ρ values (n = 10 subtypes) from CollecTRI-87 versus ChIP-anchored regulons. Pearson's correlation coefficients are indicated. (C) Empirical null distribution of Pearson's r values from 200 randomly sampled target subsets of equal size. (D) Magnified view of CREB1 peak proximity to the CREM transcription start site (TSS, ±2 kb window). (E) Venn diagram showing the nested structure of the three regulon definitions. Dataset source: panels A1-A4 and D, ENCODE CREB1 ChIP-seq, HepG2 (ENCSR000EZQ) and GM12878 (ENCSR000EHM); panels B, C and E, integrative analyses of the single-cell RNA-seq cohorts (GSE125449 + GSE151530) with the above ChIP-seq-anchored target sets and the CollecTRI prior-knowledge network.

**Figure 6:**
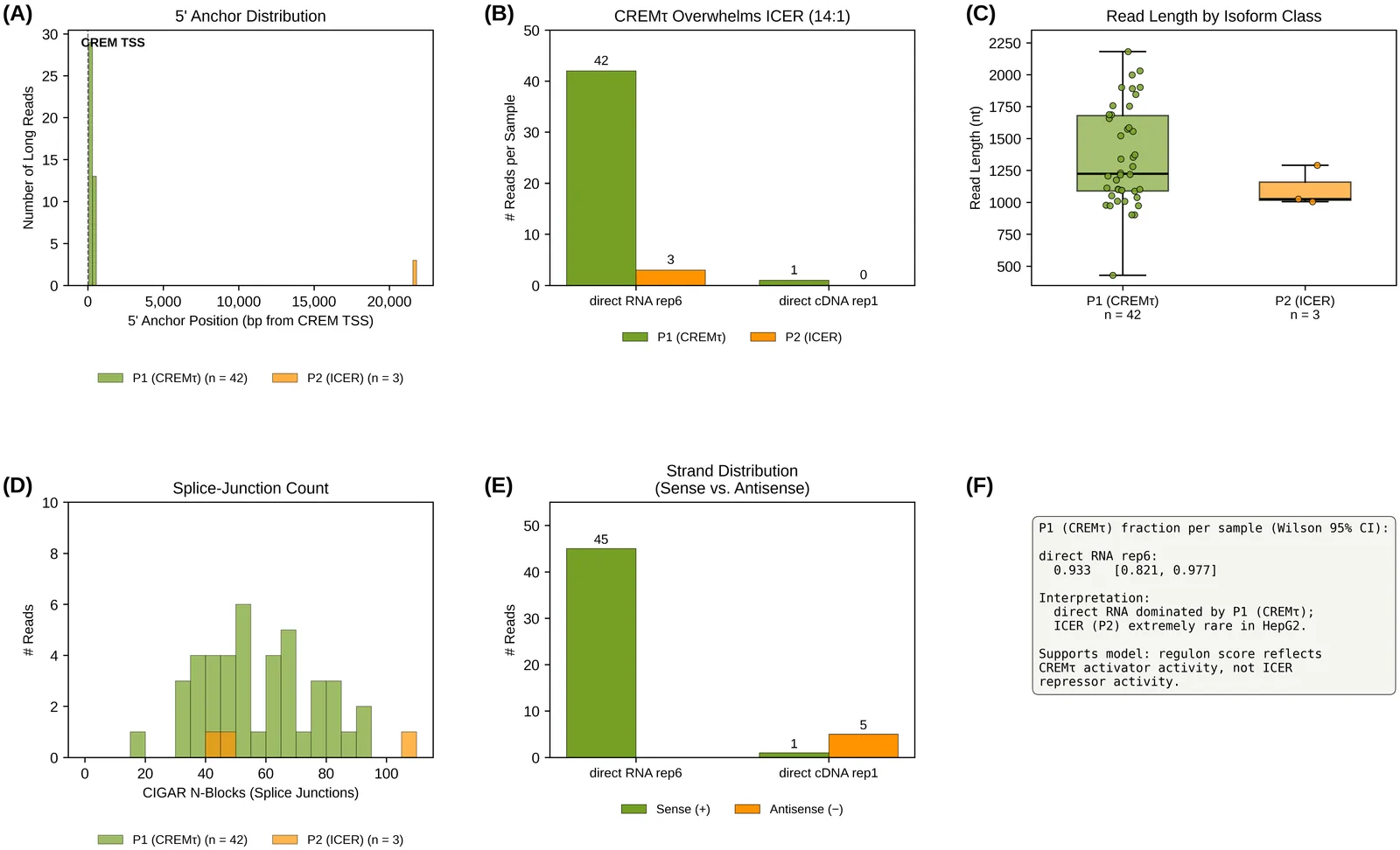
** CREM isoform usage in HepG2 cells from long-read direct RNA sequencing (direct RNA replicate 6: primary data; direct cDNA replicate 1: strand-bias control).** (A) Histogram of 5'-end positions of sense-strand reads across the CREM locus (TSS -5 kb to +30 kb) in SG-NEx HepG2 direct RNA data. Shaded regions indicate P1 (CREMτ) and P2 (ICER) promoter windows. (B) Bar plot showing the 14:1 P1:P2 isoform ratio, with 95% Wilson confidence interval for the P1 proportion. (C-D) Distributions of read length (C) and intron count (D) for P1 and P2 read classes. (E) Strand-bias diagnostic comparing sense/antisense read counts between the primary direct RNA library (replicate 6) and the direct cDNA library (replicate 1), the latter serving exclusively as the strand-bias control. (F) Summary of the P1 (CREMτ) fraction with its 95% Wilson confidence interval (0.933 [0.821, 0.977] in direct RNA replicate 6) and its interpretation for the regulon model. Dataset source: all primary quantifications (panels A-D and F) are derived from SG-NEx HepG2 direct RNA sequencing replicate 6; the direct cDNA replicate 1 library (panel E only) served solely as the strand-bias control and was not used for isoform quantification.

**Figure 7:**
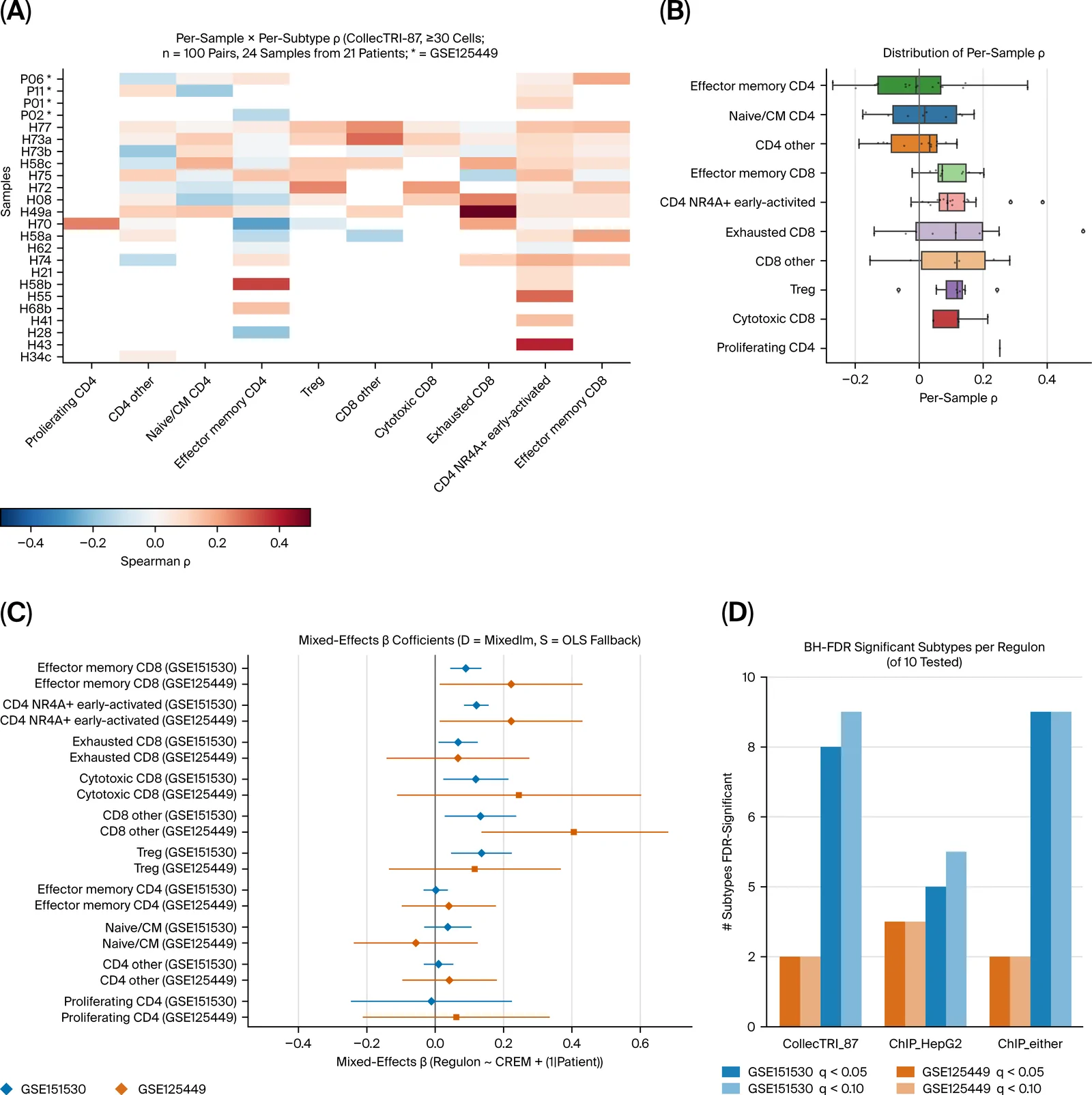
** Patient-level robustness of subtype-specific dissociation.** (A) Heatmap of per-sample per-subtype Spearman ρ values for pairs with ≥30 cells (n = 100 pairs across 24 samples from 21 patients). (B) Box plot of per-sample ρ values per subtype, ordered by median value. (C) Forest plot of β coefficients from linear mixed-effects models fit separately per cohort and subtype (regulon score ~ CREM expression + (1|patient)). (D) Bar plot of Benjamini-Hochberg false discovery rate (FDR)-significant subtype counts per regulon at q < 0.05 and q < 0.10 thresholds. Dataset source (panels A-D): single-cell RNA-seq, GSE125449 + GSE151530.

**Figure 8:**
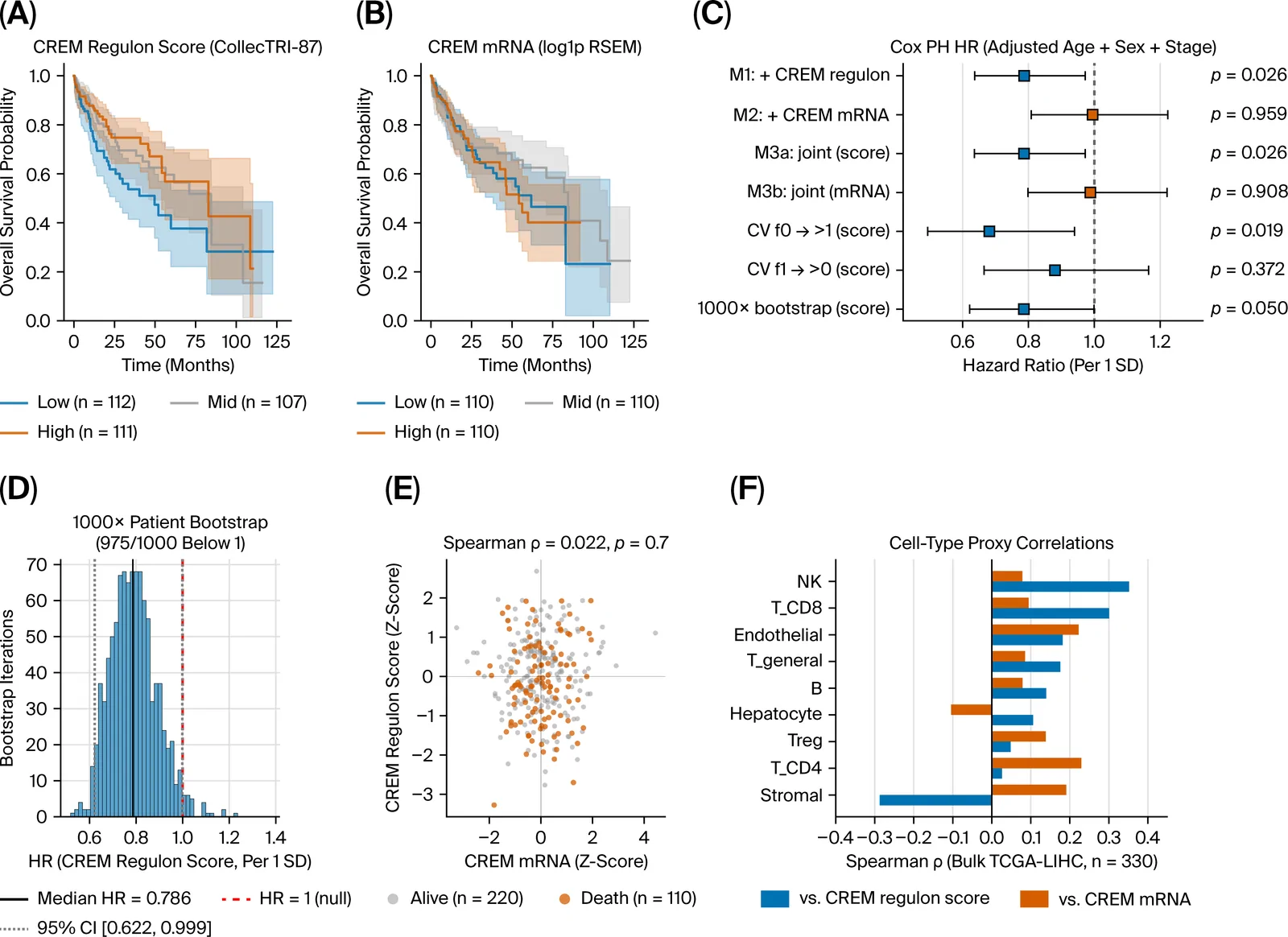
** Prognostic association of CREM regulon activity in TCGA-LIHC cohort (n = 330 patients, 110 events).** (A-B) Kaplan-Meier curves of overall survival (OS) stratified by tertiles of CollecTRI-87 regulon score (A) and CREM mRNA expression (B). Log-rank test p-values are indicated. Tertile cutoffs were computed on unique values, yielding slightly unequal group sizes for the regulon score (Low/Mid/High n = 112/107/111) and exactly equal thirds for mRNA (n = 110 each). (C) Forest plot of adjusted Cox proportional-hazards model results (covariates: age, sex, AJCC stage), held-out 2-fold cross-validation results (CV f0→1 and f1→0), and the 1000× patient-bootstrap summary. (D) Distribution of hazard ratios (HRs) from 1000 rounds of patient-resampling bootstrap for regulon score. (E) Scatter plot of CREM mRNA versus CollecTRI-87 regulon score (z-scores) across the 330 TCGA-LIHC primary tumors, colored by vital status (Alive n = 220; Death n = 110); Spearman ρ = 0.022, p = 0.7. (F) Spearman correlations of regulon score and CREM mRNA with cell-type marker panels. Dataset source (panels A-F): TCGA-LIHC bulk RNA-seq with clinical annotation (n = 330 primary tumors, 110 events).

**Figure 9:**
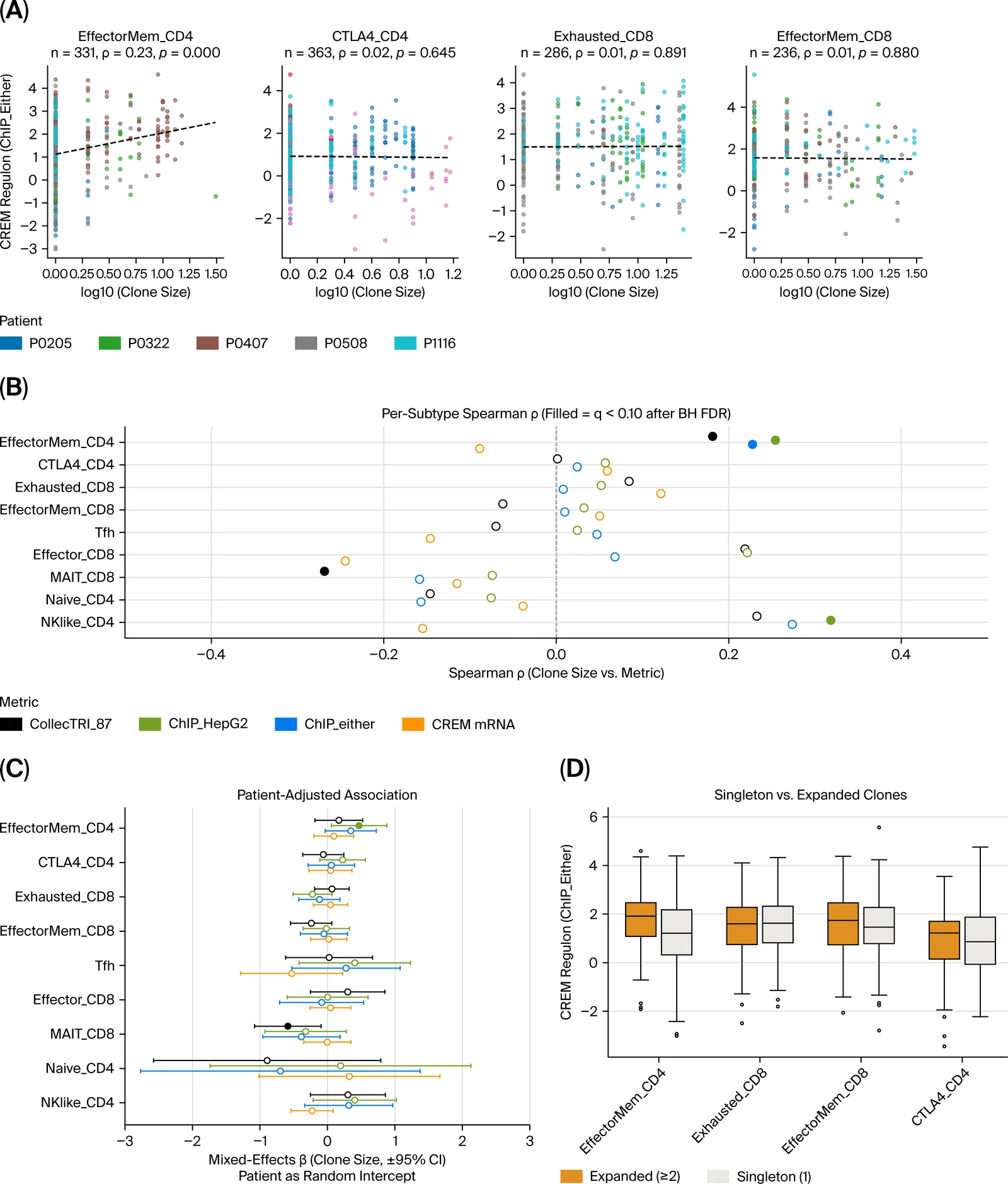
** Association between CREM regulon activity and TCR clonal expansion in GSE98638.** (A) Scatter plots of log10(CDR3β clone size) versus ChIP_either-42 regulon score for the four most abundant tumor-infiltrating T-cell subtypes, colored by patient. Least-squares fit lines and Spearman ρ values are shown. (B) Forest plot of per-subtype Spearman ρ values for log10(clone size) versus CollecTRI-87 regulon score, with BH-adjusted 95% confidence intervals (CIs) and q-values. (C) Forest plot of β coefficients from mixed-effects models with patient as random intercept, per subtype per regulon. (D) Box plot comparing CollecTRI-87 regulon z-scores between singleton (clone size = 1) and expanded (clone size ≥ 2) clones, per subtype. Wilcoxon rank-sum test p-values are indicated. Dataset source (panels A-D): single-cell RNA-seq with paired TCR sequencing of HCC-infiltrating T cells, GSE98638 (5 patients; 1,914 tumor-infiltrating T cells, of which 1,633 carry a paired TCRβ clonotype).

**Figure 10:**
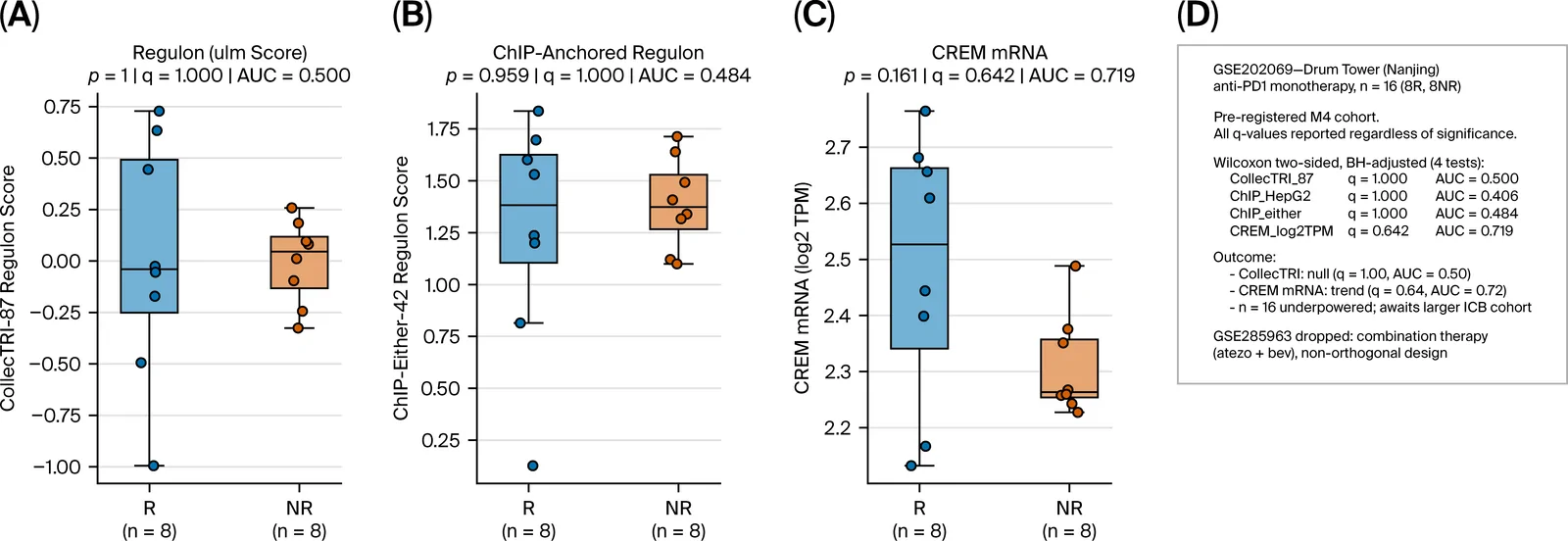
** Association of CREM metrics with anti-PD-1 response in GSE202069 (n = 16 anti-PD-1-treated patients).** (A-C) Box plots of CollecTRI-87 regulon score (A), ChIP_either-42 regulon score (B), and CREM mRNA (log2 TPM) (C) in responders (R; PR/CR, n = 8) versus non-responders (NR; SD/PD, n = 8). Two-sided Wilcoxon rank-sum p-values, BH-adjusted q-values (4 tests), and AUC values are indicated above each panel. (D) Summary of the pre-registered cohort analysis: BH-adjusted q-values and AUCs for all four CREM metrics (CollecTRI-87, ChIP_HepG2-27, ChIP_either-42, and CREM mRNA), and the rationale for dropping the GSE285963 cohort (combination atezolizumab-bevacizumab therapy; non-orthogonal design). Dataset source (panels A-D): anti-PD-1-treated HCC cohort bulk RNA-seq with response annotation, GSE202069 (n = 16 anti-PD-1-treated patients). Reference 50 deposited the GSE202069 series as part of an HCC metabolic-subtype study and is not the original trial publication for this checkpoint cohort.

**Table 1:** Per-subtype dissociation of CREM mRNA from CREM-regulon (CollecTRI-87) activity. Ten subtypes meeting the pre-specified representation threshold (≥30 cells, ≥3 patients contributing ≥10 cells each, and ≥1 such patient per cohort) are shown (18,342 cells in total); NK-like T (135 cells), Proliferating CD8 (92), and Naive/CM CD8 (73) were excluded for insufficient GSE125449 representation, and 23 cells remained unclassified, together accounting for the full 18,665-cell atlas.

Subtype	ρ (95% CI)	p	q (BH)	n cells	Dissociation	I² (%)
**Proliferating CD4**	-0.009 [-0.145, 0.126]	0.8928	0.8928	213	0.991	0.0
**CD4 other**	0.051 [0.012, 0.090]	0.0108	0.0135	2508	0.949	6.3
**Naive/CM CD4**	0.066 [0.004, 0.128]	0.0379	0.0421	984	0.934	0.0
**Effector memory CD4**	0.099 [0.067, 0.131]	<0.0001	<0.0001	3684	0.901	72.2
**Treg**	0.126 [0.053, 0.197]	0.0007	0.0011	719	0.874	0.0
**CD8 other**	0.139 [0.065, 0.212]	0.0002	0.0004	690	0.861	76.2
**Cytotoxic CD8**	0.152 [0.076, 0.226]	0.0001	0.0002	655	0.848	0.0
**Exhausted CD8**	0.162 [0.112, 0.211]	<0.0001	<0.0001	1488	0.839	0.0
**CD4 NR4A+ early-activated**	0.176 [0.147, 0.204]	<0.0001	<0.0001	4441	0.824	65.1
**Effector memory CD8**	0.180 [0.145, 0.215]	<0.0001	<0.0001	2960	0.820	0.0

Cross-cohort Fisher-z meta-analysis (cohort fixed-effects, normal-approximation 95% CI) across GSE125449 and GSE151530. Subtypes ordered by ascending ρ. Dissociation index = 1 - |ρ|. Full per-cohort statistics, bootstrap CIs, mixed-effects model output, and ChIP_HepG2/ChIP_either regulon variants in Table S4. q-values BH-adjusted across 10 subtypes. Abbreviations: ρ, Spearman's rank correlation coefficient; CI, confidence interval; q (BH), Benjamini-Hochberg-adjusted q-value; n cells, number of cells; I² (%), between-cohort heterogeneity statistic.

**Table 2:** TCGA-LIHC adjusted Cox proportional-hazards regression for CREM-regulon score vs CREM mRNA (n = 330 primary tumors, 110 events).

Model	n_patients	n_events	Variable	HR	HR_CI_lower	HR_CI_upper	p_value	Coef	se	Concordance	AIC	Concordance_test
**Baseline (age+sex+stage)**	330	110	(no focus)							0.6268	1095	
**+ CREM regulon score (CollecTRI-87)**	330	110	CREM_score_z	0.7869	0.6368	0.9724	0.02648	-0.2396	0.108	0.6373	1092	
**+ CREM mRNA (log1p RSEM)**	330	110	CREM_log1p_z	0.9945	0.8086	1.223	0.9587	-0.005475	0.1056	0.6273	1097	
**Joint: + score + mRNA**	330	110	CREM_score_z	0.7864	0.636	0.9723	0.02648	-0.2403	0.1083	0.638	1094	
**Joint: + score + mRNA**	330	110	CREM_log1p_z	0.9876	0.7989	1.221	0.9083	-0.01245	0.1082	0.638	1094	
**Held-out: train fold 0, test fold 1**	161	52	CREM_score_z	0.6816	0.4942	0.94	0.01942	-0.3833	0.164	0.6911	443	0.5934
**Held-out: train fold 1, test fold 0**	169	58	CREM_score_z	0.8804	0.6656	1.165	0.3724	-0.1273	0.1427	0.6233	504.4	0.642
**1000x patient bootstrap**	330	110	CREM_score_z	0.7859	0.6218	0.999	0.05					

## Data Availability

All data analyzed in this study are publicly available. Single-cell RNA-seq: GSE125449 and GSE151530 from GEO; GSE98638 (HCC TCR-paired scRNA-seq, Zheng et al. 2017) from GEO; GSE202069 (anti-PD-1 HCC bulk RNA-seq) from GEO. ChIP-seq: ENCSR000EZQ (HepG2 CREB1) and ENCSR000EHM (GM12878 CREB1) from the ENCODE Portal. Long-read RNA-seq: SG-NEx HepG2 direct RNA replicate 6 and direct cDNA replicate 1 from the SG-NEx S3 bucket; DRR465281 from the DDBJ/ENA. Additionally screened public T-cell long-read datasets (Table S3): TRAILS [29] (DRA016285; DDBJ/ENA); Woolley et al. 2025 [30] (GSE229971) and Woolley et al. 2022 [31] (GSE202327) from GEO; E-MTAB-10797 [32] (ERP130787; ERR6357499, ERR6357500, ERR6357501) from ENA/ArrayExpress; Fanari et al. 2025 [33] (SRP520260; SRR29847356, SRR29847359) from SRA. TCGA-LIHC primary-tumor RNA-seq and clinical data from Broad GDAC Firehose (LIHC.rnaseq v2 RSEM-normalized expression; LIHC.Clinical_Pick_Tier1.Level_4.2016012800.0.0). The analysis code used in this study is available from the corresponding author upon reasonable request.

## Abbreviations

AJCC: American Joint Committee on Cancer
ATF-1: Activating Transcription Factor 1
AUC: Area Under the Curve
BH-FDR: Benjamini-Hochberg False Discovery Rate
bZIP: Basic Leucine Zipper
cAMP: Cyclic Adenosine Monophosphate
CDR3: Complementarity-Determining Region 3
ChIP-seq: Chromatin Immunoprecipitation Sequencing
CI: Confidence Interval
CM: Central Memory
CollecTRI: Literature-Curated Transcriptomic Regulon Database
CRE: cAMP Response Element
CREB1: cAMP Response Element-Binding Protein 1
CREM: cAMP-Responsive Element Modulator
direct cDNA: Direct cDNA Sequencing
direct RNA: Direct RNA Sequencing
ENCODE: Encyclopedia of DNA Elements
FDR: False Discovery Rate
HCC: Hepatocellular Carcinoma
HR: Hazard Ratio
HVGs: Highly Variable Genes
iCCA: Intrahepatic Cholangiocarcinoma
ICER: Inducible cAMP Early Repressor
IDR: Irreproducible Discovery Rate
iLISI: Integration Local Inverse Simpson's Index
kBET: k-nearest neighbor Batch Effect Test
MDSCs: Myeloid-Derived Suppressor Cells
NK: Natural Killer cell
NR4A: Nuclear Receptor Subfamily 4 Group A
OS: Overall Survival
PGE2: Prostaglandin E2
PKA: Protein Kinase A
RSEM: RNA-Seq by Expectation-Maximization
SCENIC: Single-Cell Regulatory Network Inference and Clustering
scRNA-seq: Single-Cell RNA Sequencing
scVI: Single-Cell Variational Inference
SD: Standard Deviation
TAMs: Tumor-Associated Macrophages
TCGA: The Cancer Genome Atlas
TCGA-LIHC: Liver Hepatocellular Carcinoma
TCR: T-Cell Receptor
TILs: Tumor-Infiltrating Lymphocytes
Treg: Regulatory T cell
TREM2: Triggering Receptor Expressed on Myeloid Cells 2
TSS: Transcription Start Site
UMAP: Uniform Manifold Approximation and Projection
UMI: Unique Molecular Identifier
