# Supplementary material for: State-Dependent Decoupling of a CREM-Centric cAMP-Responsive Regulon and Survival in Hepatocellular Carcinoma
Source: Int J Med Sci. 2026 Oct 2;23(11):3464–82. doi: 10.7150/ijms.140851 (PMC13685465; doi:10.7150/ijms.140851)
Supplement: Supplementary file 1 [file ijmsv23p3464s1.pdf]

Supplementary Material

State-Dependent Decoupling of a CREM-Centric cAMP-Responsive Regulon and Survival in Hepatocellular Carcinoma

Hao Ling, Yanzhu Hu — Int J Med Sci manuscript 140851u

Supplementary Figures

Figure S1. Cohort quality control and integration (A) Bar chart of T-cell counts per patient. (B) Percent mitochondrial reads per cell, (C) number of detected genes per cell, and (D) Distribution of log10 unique molecular identifier (UMI) counts per cell, stratified by cohort. (E) Stacked bar plot of cohort composition per scVI Leiden cluster (resolution 0.3). UMAP projection colored by cohort before (F) and after (G) scVI integration. (H) iLISI head-to-head comparison of Harmony and scVI integration across multiple neighborhood sizes, computed on a fixed 3,000-cell stratified subsample. Dataset source (panels A-H): single-cell RNA-seq, GSE125449 + GSE151530.

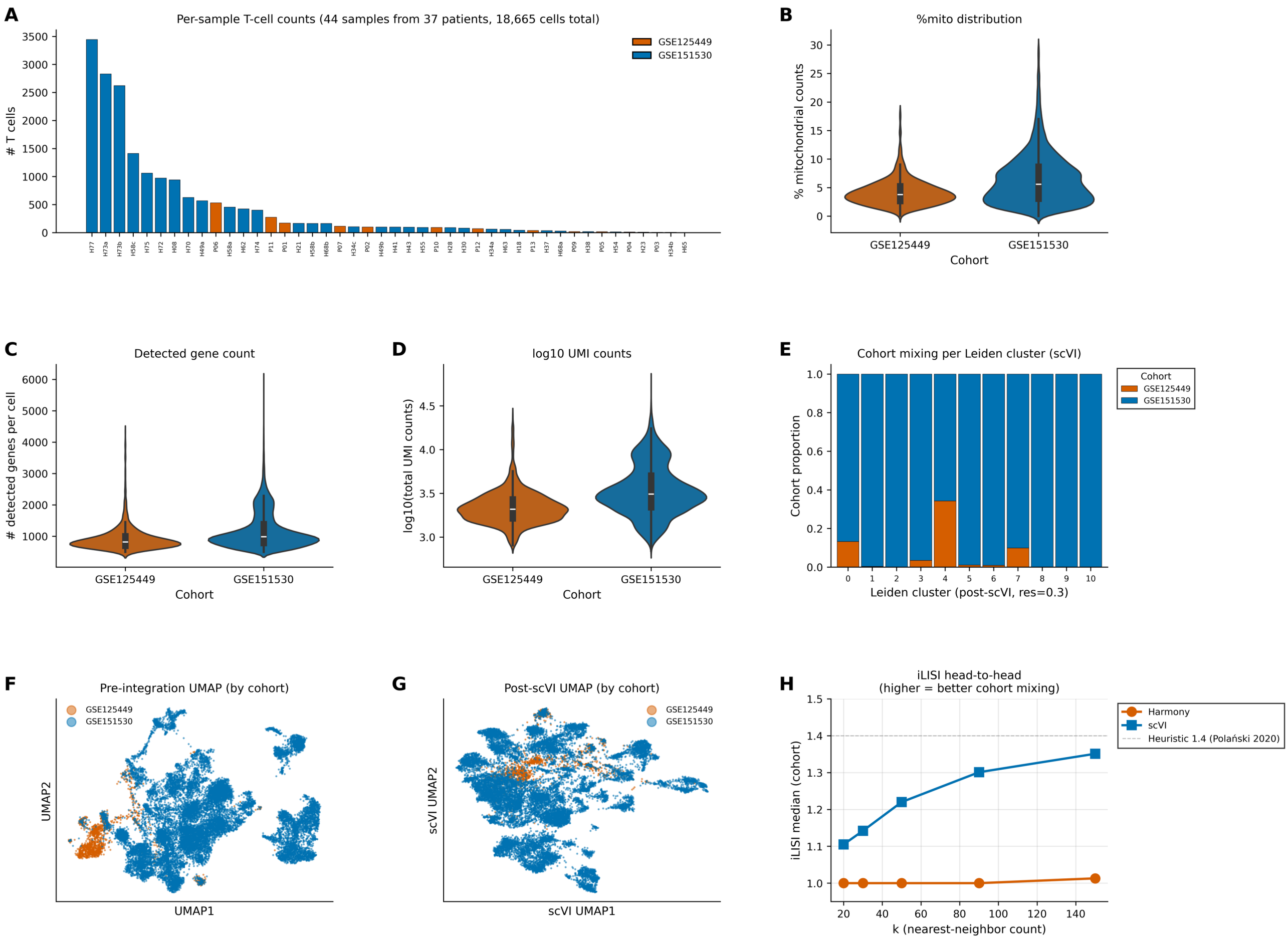

Figure S2. T-cell subtype annotation quality control (A) Marker-score heatmap of subtype definitions (rows) versus canonical marker genes (columns), Z-scored. (B) Confusion matrix comparing scVI Leiden clusters (resolution 0.3) versus marker-gated subtype annotations (row-normalized). (C) Grouped bar plot of per-subtype cell counts stratified by cohort. (D) scVI-integrated UMAP colored by marker-gated subtype calls. Dataset source (panels A-D): single-cell RNA-seq, GSE125449 + GSE151530.

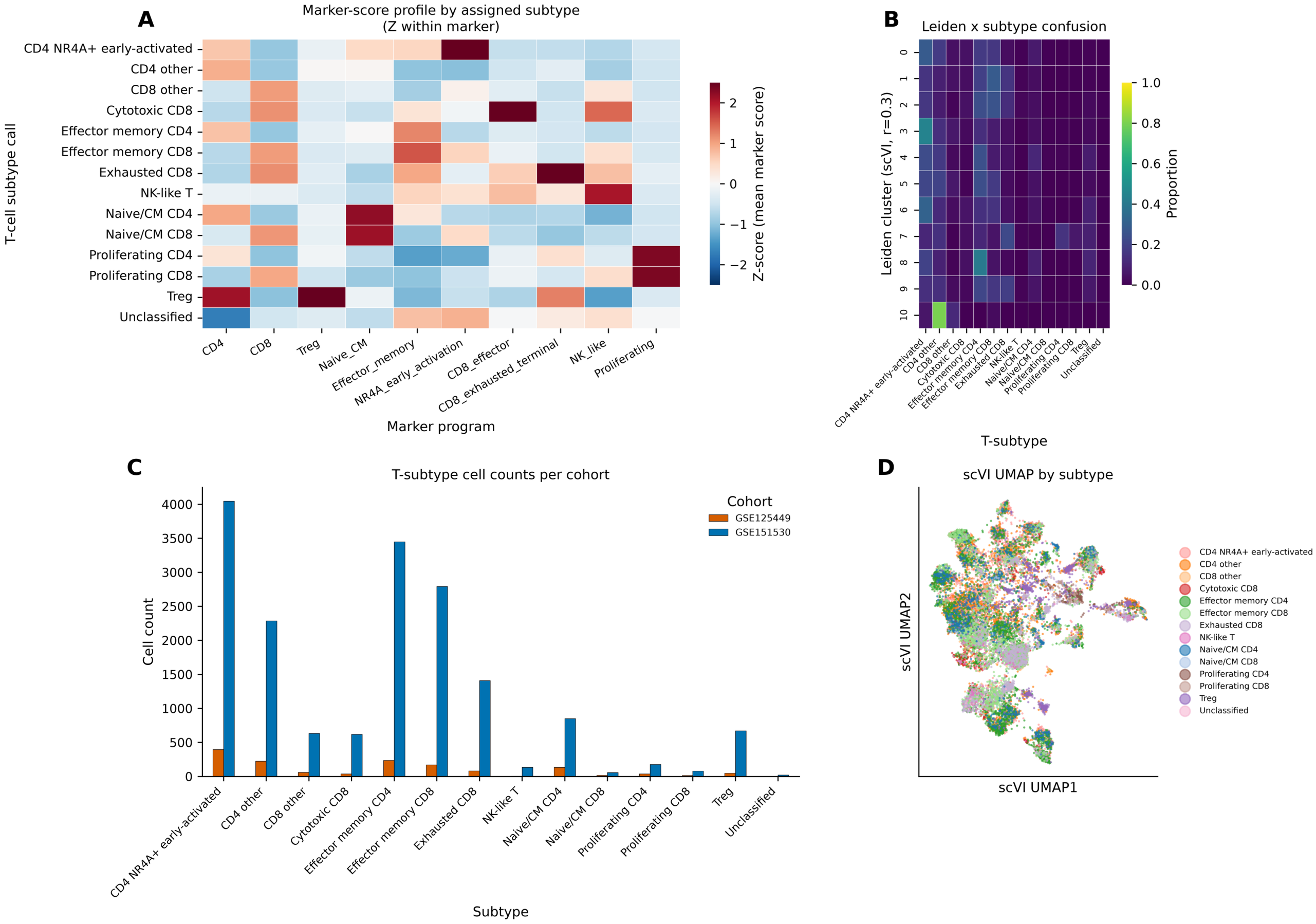

Figure S3. Regulon definition sensitivity analysis (A) Bar plot of target gene counts for the three regulon definitions. (B) Bar plot comparing pooled-cohort Spearman ρ values across regulons, for all cells and CREM^{+}-only cells. (C) Scatter plot of per-cohort ρ values and (D) box plot of per-subtype meta-ρ values across three regulons. (E) Scatter plot comparing CollecTRI-87 and ChIP-anchored regulon ρ values computed per cohort × subtype (n = 20 cohort × subtype pairs per comparison), a complementary aggregation level to the per-subtype meta-ρ comparison in Figure 5B. Dataset source (panels A-E): single-cell RNA-seq, GSE125449 + GSE151530; regulon definitions derive from the CollecTRI prior-knowledge network and ENCODE CREB1 ChIP-seq (ENCSR000EZQ, ENCSR000EHM).

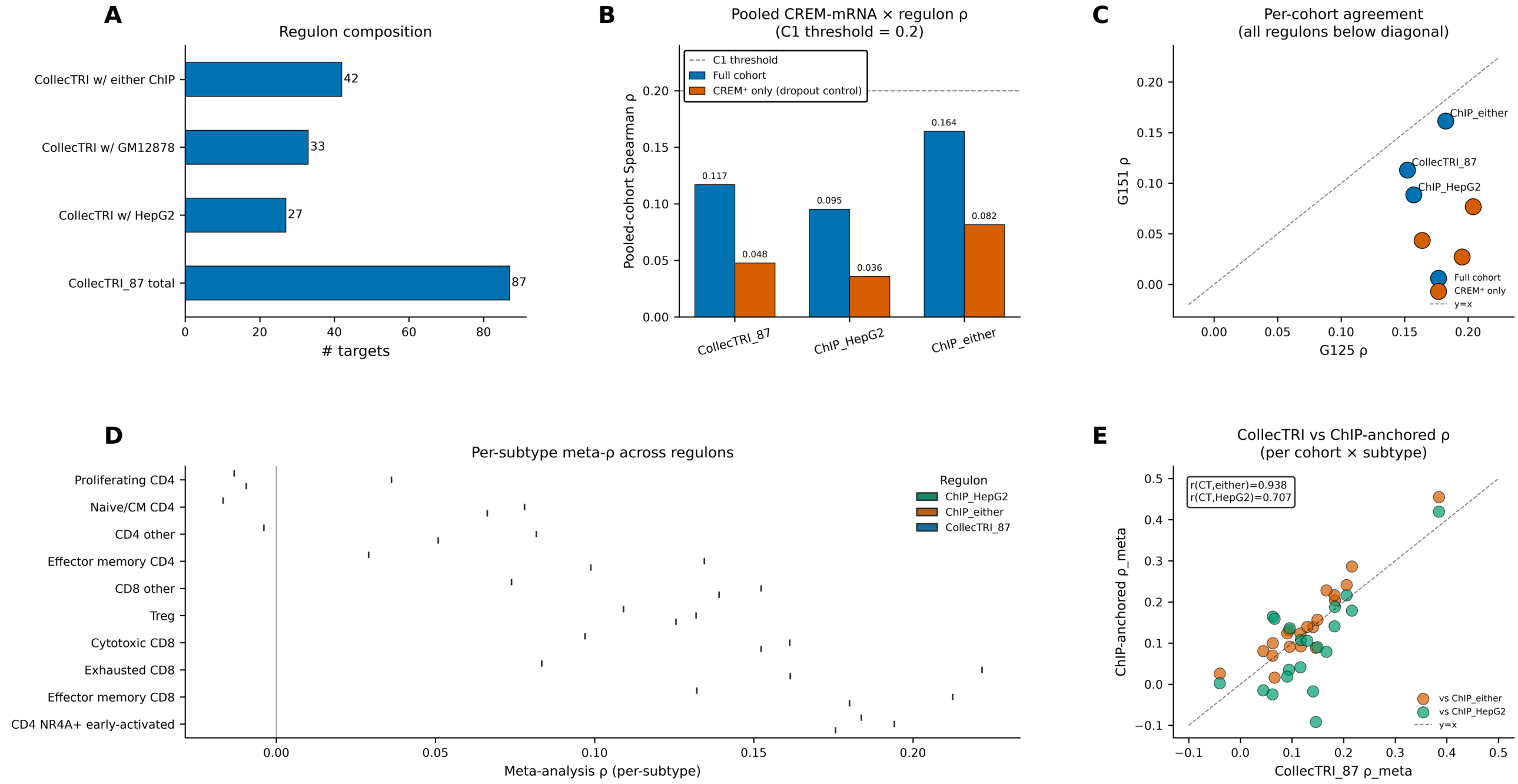

Figure S4. Long-read per-read details and direct cDNA control (A) Scatter plot of per-read 5'-anchor position versus read length, colored by strand. (B) Bar plot of sense/antisense read counts for direct RNA and direct cDNA libraries. Box plots of read length (C) and intron count (D) for P1 and P2 read classes, the only sense-read classes in the final classification. (E) Summary panel with full read-count breakdown. Dataset source (panels A-E): SG-NEx HepG2 long-read RNA-seq, direct RNA replicate 6 and direct cDNA replicate 1.

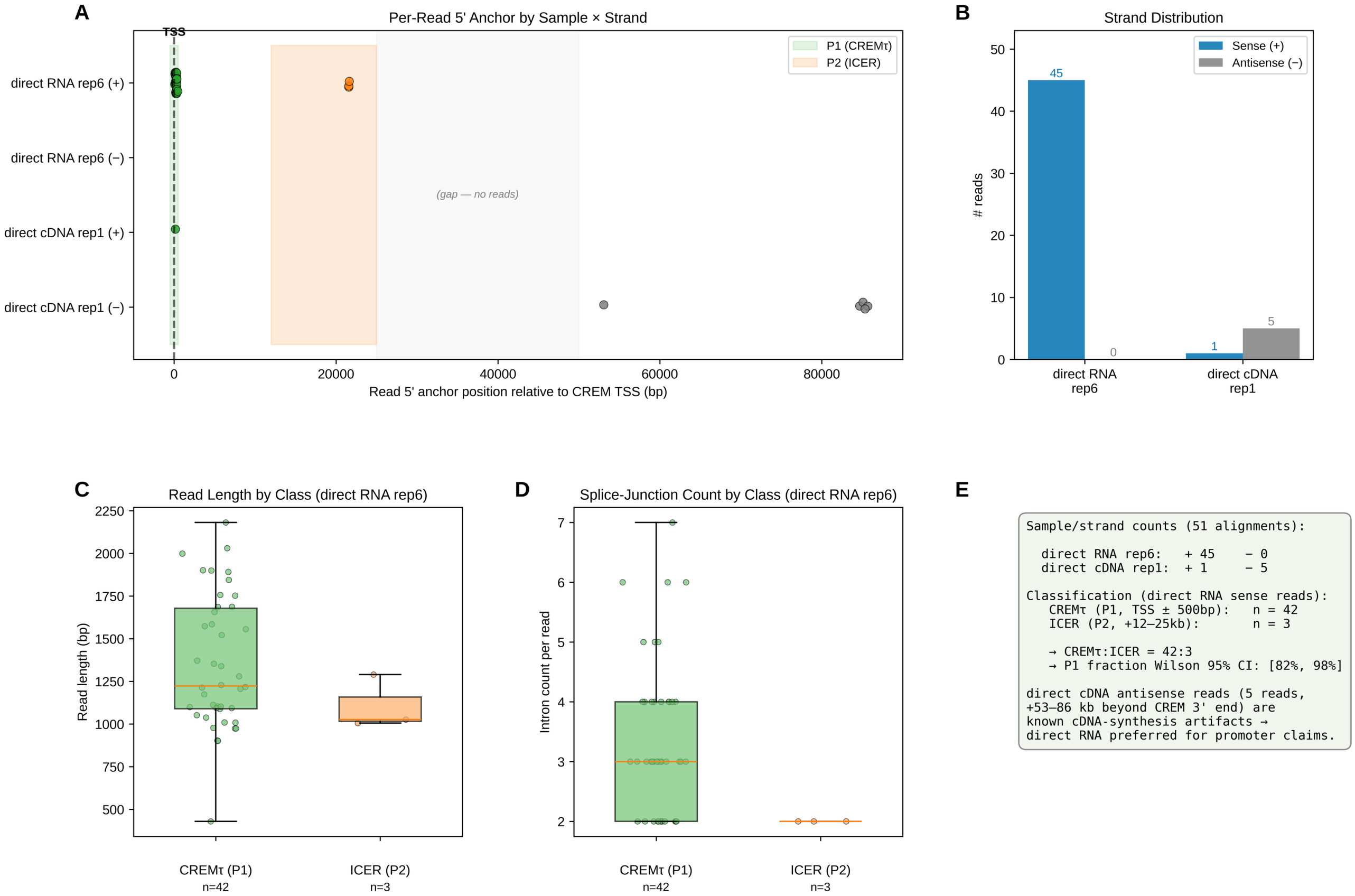

Supplementary Tables

Table S1. HCC-only sensitivity analysis (excluding the 5 iCCA patients from GSE125449): pooled and per-subtype meta-analytic correlations, and GSE125449 mixed-effects model outputs.

Table S1 (part 1 of 3)

section	regulon	scope	n_cells_orig	rho_orig	p_orig	n_cells_hcc_only	rho_hcc_only	p_hcc_only	d_rho	subtype	n_cells_G125_orig
C1_pooled	CollecTRI_87	full	18665	0.117	6.24e-58	17768	0.1143	1.019e-52	-0.00277		
C1_pooled	CollecTRI_87	CREMpos	10893	0.04778	6.068e-07	10474	0.04639	2.041e-06	-0.001393		
C1_pooled	ChIP_HepG2	full	18665	0.09538	5.64e-39	17768	0.09134	3.128e-34	-0.004039		
C1_pooled	ChIP_HepG2	CREMpos	10893	0.03589	0.0001791	10474	0.03443	0.0004247	-0.001463		
C1_pooled	ChIP_either	full	18665	0.1642	5.738e-113	17768	0.1629	6.827e-106	-0.001339		
C1_pooled	ChIP_either	CREMpos	10893	0.08166	1.388e-17	10474	0.08281	2.096e-17	0.001148		
C2_subtype_meta	ChIP_HepG2									CD4 NR4A+ early-activated	395
C2_subtype_meta	ChIP_HepG2									CD4 other	223
C2_subtype_meta	ChIP_HepG2									CD8 other	58
C2_subtype_meta	ChIP_HepG2									Cytotoxic CD8	38
C2_subtype_meta	ChIP_HepG2									Effector memory CD4	236
C2_subtype_meta	ChIP_HepG2									Effector memory CD8	168
C2_subtype_meta	ChIP_HepG2									Exhausted CD8	80
C2_subtype_meta	ChIP_HepG2									Naive/CM CD4	134
C2_subtype_meta	ChIP_HepG2									Proliferating CD4	37
C2_subtype_meta	ChIP_HepG2									Treg	48
C2_subtype_meta	ChIP_either									CD4 NR4A+ early-activated	395
C2_subtype_meta	ChIP_either									CD4 other	223
C2_subtype_meta	ChIP_either									CD8 other	58
C2_subtype_meta	ChIP_either									Cytotoxic CD8	38
C2_subtype_meta	ChIP_either									Effector memory CD4	236
C2_subtype_meta	ChIP_either									Effector memory CD8	168
C2_subtype_meta	ChIP_either									Exhausted CD8	80
C2_subtype_meta	ChIP_either									Naive/CM CD4	134
C2_subtype_meta	ChIP_either									Proliferating CD4	37
C2_subtype_meta	ChIP_either									Treg	48
C2_subtype_meta	CollecTRI_87									CD4 NR4A+ early-activated	395
C2_subtype_meta	CollecTRI_87									CD4 other	223
C2_subtype_meta	CollecTRI_87									CD8 other	58
C2_subtype_meta	CollecTRI_87									Cytotoxic CD8	38
C2_subtype_meta	CollecTRI_87									Effector memory CD4	236
C2_subtype_meta	CollecTRI_87									Effector memory CD8	168
C2_subtype_meta	CollecTRI_87									Exhausted CD8	80
C2_subtype_meta	CollecTRI_87									Naive/CM CD4	134
C2_subtype_meta	CollecTRI_87									Proliferating CD4	37
C2_subtype_meta	CollecTRI_87									Treg	48
C2_mixed_model_G125	CollecTRI_87									CD4 NR4A+ early-activated	
C2_mixed_model_G125	ChIP_HepG2									CD4 NR4A+ early-activated	
C2_mixed_model_G125	ChIP_either									CD4 NR4A+ early-activated	
C2_mixed_model_G125	CollecTRI_87									CD4 other	

section	regulon	scope	n_cells_orig	rho_orig	p_orig	n_cells_hcc_only	rho_hcc_only	p_hcc_only	d_rho	subtype	n_cells_G125_orig
C2_mixed_model_G125	ChIP_HepG2									CD4 other	
C2_mixed_model_G125	ChIP_either									CD4 other	
C2_mixed_model_G125	CollecTRI_87									CD8 other	
C2_mixed_model_G125	ChIP_HepG2									CD8 other	
C2_mixed_model_G125	ChIP_either									CD8 other	
C2_mixed_model_G125	CollecTRI_87									Cytotoxic CD8	
C2_mixed_model_G125	ChIP_HepG2									Cytotoxic CD8	
C2_mixed_model_G125	ChIP_either									Cytotoxic CD8	
C2_mixed_model_G125	CollecTRI_87									Effector memory CD4	
C2_mixed_model_G125	ChIP_HepG2									Effector memory CD4	
C2_mixed_model_G125	ChIP_either									Effector memory CD4	
C2_mixed_model_G125	CollecTRI_87									Effector memory CD8	
C2_mixed_model_G125	ChIP_HepG2									Effector memory CD8	
C2_mixed_model_G125	ChIP_either									Effector memory CD8	
C2_mixed_model_G125	CollecTRI_87									Exhausted CD8	
C2_mixed_model_G125	ChIP_HepG2									Exhausted CD8	
C2_mixed_model_G125	ChIP_either									Exhausted CD8	
C2_mixed_model_G125	CollecTRI_87									Naive/CM CD4	
C2_mixed_model_G125	ChIP_HepG2									Naive/CM CD4	
C2_mixed_model_G125	ChIP_either									Naive/CM CD4	
C2_mixed_model_G125	CollecTRI_87									Proliferating CD4	
C2_mixed_model_G125	ChIP_HepG2									Proliferating CD4	
C2_mixed_model_G125	ChIP_either									Proliferating CD4	
C2_mixed_model_G125	CollecTRI_87									Treg	
C2_mixed_model_G125	ChIP_HepG2									Treg	
C2_mixed_model_G125	ChIP_either									Treg	

Table S1 (part 2 of 3)

section	regulon	scope	n_patients_G125_orig	rho_full_G125_orig	n_cells_G125_hcc	n_patients_G125_hcc	rho_full_G125_hcc	rho_full_G151_orig	rho_meta_orig	q_meta_BH_orig	rho_meta_hcc	q_meta_BH_hcc	d_rho_meta
C1_pooled	CollecTRI_87	full											
C1_pooled	CollecTRI_87	CREMpos											

section	regulon	scope	n_patients_G125_orig	rho_full_G125_orig	n_cells_G125_hcc	n_patients_G125_hcc	rho_full_G125_hcc	rho_full_G151_orig	rho_meta_orig	q_meta_BH_orig	rho_meta_hcc	q_meta_BH_hcc	d_rho_meta
C1_pooled	ChIP_HepG2	full											
C1_pooled	ChIP_HepG2	CREMpos											
C1_pooled	ChIP_either	full											
C1_pooled	ChIP_either	CREMpos											
C2_subtype_meta	ChIP_HepG2		11	0.1363	135	7	0.17	0.1884	0.1838	3.343e-34	0.1878	1.179e-33	0.003987
C2_subtype_meta	ChIP_HepG2		11	0.1077	83	7	0.16	-0.01466	-0.003863	0.8494	-0.008696	0.7473	-0.004832
C2_subtype_meta	ChIP_HepG2		9	0.4198	15	6	0.4351	0.04135	0.07389	0.08813	0.04928	0.3025	-0.0246
C2_subtype_meta	ChIP_HepG2		10	0.216	18	7	-0.1467	0.09013	0.09703	0.0263	0.08449	0.06733	-0.01254
C2_subtype_meta	ChIP_HepG2		12	0.179	97	7	0.2945	0.0187	0.02897	0.1127	0.02626	0.197	-0.002711
C2_subtype_meta	ChIP_HepG2		11	-0.0172	41	6	-0.1157	0.1408	0.1321	2.592e-12	0.1374	9.832e-13	0.005311
C2_subtype_meta	ChIP_HepG2		8	0.1646	59	5	0.1544	0.07891	0.0834	0.004301	0.08182	0.005739	-0.001579
C2_subtype_meta	ChIP_HepG2		11	0.03515	46	6	0.06679	-0.02468	-0.01666	0.7528	-0.02025	0.682	-0.003591
C2_subtype_meta	ChIP_HepG2		9	-0.09177	24	6	-0.2085	0.002297	-0.01319	0.8494	-0.02085	0.7715	-0.007657
C2_subtype_meta	ChIP_HepG2		10	0.1592	17	5	0.07371	0.1057	0.1091	0.008641	0.105	0.01479	-0.004056
C2_subtype_meta	ChIP_either		11	0.09136	135	7	0.1335	0.204	0.1942	3.22e-38	0.2018	6.551e-39	0.007585
C2_subtype_meta	ChIP_either		11	0.09244	83	7	0.08669	0.08065	0.08169	7.029e-05	0.08086	0.0001641	-0.0008326
C2_subtype_meta	ChIP_either		9	0.4553	15	6	0.5681	0.1233	0.1523	8.533e-05	0.1329	0.0008915	-0.01939
C2_subtype_meta	ChIP_either		10	0.2416	18	7	-0.1843	0.1567	0.1613	6.779e-05	0.1486	0.0002883	-0.01267
C2_subtype_meta	ChIP_either		12	0.2863	97	7	0.2558	0.1239	0.1345	5.647e-16	0.1275	6.009e-14	-0.007004
C2_subtype_meta	ChIP_either		11	0.1394	41	6	-0.1419	0.2168	0.2125	4.455e-31	0.2121	1.13e-29	-0.0003943
C2_subtype_meta	ChIP_either		8	0.09981	59	5	0.04174	0.2282	0.2217	1.355e-17	0.2212	2.692e-17	-0.0004159
C2_subtype_meta	ChIP_either		11	0.1312	46	6	0.1723	0.0697	0.07798	0.01615	0.07471	0.02839	-0.003273
C2_subtype_meta	ChIP_either		9	0.08882	24	6	-0.03658	0.02583	0.0362	0.6023	0.01907	0.7905	-0.01713
C2_subtype_meta	ChIP_either		10	0.01627	17	5	0.1744	0.1396	0.1319	0.0004967	0.1403	0.000323	0.008415
C2_subtype_meta	CollecTRI_87		11	0.09562	135	7	0.1816	0.1834	0.1757	2.946e-31	0.1833	4.543e-32	0.007606
C2_subtype_meta	CollecTRI_87		11	0.1169	83	7	0.108	0.04451	0.05091	0.01352	0.04667	0.02904	-0.00424
C2_subtype_meta	CollecTRI_87		9	0.3845	15	6	0.5439	0.1163	0.1391	0.0004158	0.1254	0.002018	-0.01373
C2_subtype_meta	CollecTRI_87		10	0.2059	18	7	-0.06925	0.1492	0.1523	0.0001851	0.144	0.0005497	-0.008215
C2_subtype_meta	CollecTRI_87		12	0.2159	97	7	0.1183	0.09076	0.09882	4.565e-09	0.09149	1.203e-07	-0.007324
C2_subtype_meta	CollecTRI_87		11	0.1407	41	6	-0.1557	0.1824	0.1801	2.216e-22	0.1779	5.789e-21	-0.00213
C2_subtype_meta	CollecTRI_87		8	0.06286	59	5	0.01615	0.1668	0.1615	1.194e-09	0.1611	1.733e-09	-0.0003477
C2_subtype_meta	CollecTRI_87		11	0.09433	46	6	0.2195	0.06195	0.0663	0.04207	0.0697	0.04141	0.003405
C2_subtype_meta	CollecTRI_87		9	0.1462	24	6	0.03292	-0.04013	-0.009366	0.8928	-0.03223	0.6534	-0.02287
C2_subtype_meta	CollecTRI_87		10	0.06632	17	5	0.1843	0.1296	0.1256	0.001065	0.1307	0.0009925	0.0051
C2_mixed_model_G125	CollecTRI_87												
C2_mixed_model_G125	ChIP_HepG2												
C2_mixed_model_G125	ChIP_either												
C2_mixed_model_G125	CollecTRI_87												
C2_mixed_model_G125	ChIP_HepG2												
C2_mixed_model_G125	ChIP_either												
C2_mixed_model_G125	CollecTRI_87												
C2_mixed_model_G125	ChIP_HepG2												
C2_mixed_model_G125	ChIP_either												

section	regulon	scope	n_patients_G125_orig	rho_full_G125_orig	n_cells_G125_hcc	n_patients_G125_hcc	rho_full_G125_hcc	rho_full_G151_orig	rho_meta_orig	q_meta_BH_orig	rho_meta_hcc	q_meta_BH_hcc	d_rho_meta
C2_mixed_model_G125	CollecTRI_87												
C2_mixed_model_G125	ChIP_HepG2												
C2_mixed_model_G125	ChIP_either												
C2_mixed_model_G125	CollecTRI_87												
C2_mixed_model_G125	ChIP_HepG2												
C2_mixed_model_G125	ChIP_either												
C2_mixed_model_G125	CollecTRI_87												
C2_mixed_model_G125	ChIP_HepG2												
C2_mixed_model_G125	ChIP_either												
C2_mixed_model_G125	CollecTRI_87												
C2_mixed_model_G125	ChIP_HepG2												
C2_mixed_model_G125	ChIP_either												
C2_mixed_model_G125	CollecTRI_87												
C2_mixed_model_G125	ChIP_HepG2												
C2_mixed_model_G125	ChIP_either												
C2_mixed_model_G125	CollecTRI_87												
C2_mixed_model_G125	ChIP_HepG2												
C2_mixed_model_G125	ChIP_either												
C2_mixed_model_G125	CollecTRI_87												
C2_mixed_model_G125	ChIP_HepG2												
C2_mixed_model_G125	ChIP_either												

Table S1 (part 3 of 3)

section	regulon	scope	model_orig	beta_orig	se_orig	p_value_orig	n_patients_used_orig	model_hcc	beta_hcc	se_hcc	p_value_hcc	n_patients_used_hcc
C1_pooled	CollecTRI_87	full										
C1_pooled	CollecTRI_87	CREMpos										
C1_pooled	ChIP_HepG2	full										
C1_pooled	ChIP_HepG2	CREMpos										
C1_pooled	ChIP_either	full										
C1_pooled	ChIP_either	CREMpos										
C2_subtype_meta	ChIP_HepG2											
C2_subtype_meta	ChIP_HepG2											
C2_subtype_meta	ChIP_HepG2											
C2_subtype_meta	ChIP_HepG2											

section	regulon	scope	model_orig	beta_orig	se_orig	p_value_orig	n_patients_used_orig	model_hcc	beta_hcc	se_hcc	p_value_hcc	n_patients_used_hcc
C2_subtype_meta	ChIP_HepG2											
C2_subtype_meta	ChIP_HepG2											
C2_subtype_meta	ChIP_HepG2											
C2_subtype_meta	ChIP_HepG2											
C2_subtype_meta	ChIP_HepG2											
C2_subtype_meta	ChIP_HepG2											
C2_subtype_meta	ChIP_either											
C2_subtype_meta	ChIP_either											
C2_subtype_meta	ChIP_either											
C2_subtype_meta	ChIP_either											
C2_subtype_meta	ChIP_either											
C2_subtype_meta	ChIP_either											
C2_subtype_meta	ChIP_either											
C2_subtype_meta	ChIP_either											
C2_subtype_meta	ChIP_either											
C2_subtype_meta	ChIP_either											
C2_subtype_meta	CollecTRI_87											
C2_subtype_meta	CollecTRI_87											
C2_subtype_meta	CollecTRI_87											
C2_subtype_meta	CollecTRI_87											
C2_subtype_meta	CollecTRI_87											
C2_subtype_meta	CollecTRI_87											
C2_subtype_meta	CollecTRI_87											
C2_subtype_meta	CollecTRI_87											
C2_subtype_meta	CollecTRI_87											
C2_subtype_meta	CollecTRI_87											
C2_mixed_model_G125	CollecTRI_87		mixed_effects	0.09522	0.05771	0.09892	4	fixed_effect_OLS_fallback	0.194	0.08579	0.02537	1
C2_mixed_model_G125	ChIP_HepG2		mixed_effects	0.1376	0.06007	0.02194	4	fixed_effect_OLS_fallback	0.1945	0.08977	0.03207	1
C2_mixed_model_G125	ChIP_either		mixed_effects	0.08633	0.06382	0.1761	4	fixed_effect_OLS_fallback	0.1615	0.09961	0.1073	1
C2_mixed_model_G125	CollecTRI_87		mixed_effects	0.04159	0.06935	0.5487	7	mixed_effects	0.0143	0.1426	0.9201	4
C2_mixed_model_G125	ChIP_HepG2		mixed_effects	-0.04642	0.06815	0.4958	7	mixed_effects	-0.1727	0.123	0.1605	4
C2_mixed_model_G125	ChIP_either		mixed_effects	-0.03566	0.07812	0.6481	7	mixed_effects	-0.1978	0.1534	0.1973	4
C2_mixed_model_G125	CollecTRI_87		fixed_effect_OLS_fallback	0.4057	0.1401	0.005395	2	fixed_effect_OLS_fallback	0.4602	0.1938	0.03364	0
C2_mixed_model_G125	ChIP_HepG2		fixed_effect_OLS_fallback	0.4912	0.1363	0.0006703	2	fixed_effect_OLS_fallback	0.3969	0.2046	0.07443	0
C2_mixed_model_G125	ChIP_either		fixed_effect_OLS_fallback	0.6133	0.165	0.000467	2	fixed_effect_OLS_fallback	0.5933	0.2798	0.05379	0
C2_mixed_model_G125	CollecTRI_87		fixed_effect_OLS_fallback	0.2452	0.1812	0.1843	1	fixed_effect_OLS_fallback	-0.04727	0.2197	0.8324	0
C2_mixed_model_G125	ChIP_HepG2		fixed_effect_OLS_fallback	0.248	0.162	0.1346	1	fixed_effect_OLS_fallback	-0.07135	0.1865	0.7072	0
C2_mixed_model_G125	ChIP_either		fixed_effect_OLS_fallback	0.3266	0.2162	0.1396	1	fixed_effect_OLS_fallback	-0.1451	0.2699	0.5982	0
C2_mixed_model_G125	CollecTRI_87		mixed_effects	0.03978	0.06923	0.5656	6	mixed_effects	0.03895	0.122	0.7495	3
C2_mixed_model_G125	ChIP_HepG2		mixed_effects	0.02863	0.06486	0.6589	6	mixed_effects	0.07583	0.1019	0.4569	3

section	regulon	scope	model_orig	beta_orig	se_orig	p_value_orig	n_patients_used_orig	model_hcc	beta_hcc	se_hcc	p_value_hcc	n_patients_used_hcc
C2_mixed_model_G125	ChIP_either		mixed_effects	0.0401	0.07548	0.5952	6	mixed_effects	0.1316	0.1356	0.332	3
C2_mixed_model_G125	CollecTRI_87		mixed_effects	0.222	0.1057	0.03568	4	fixed_effect_OLS_fallback	-0.0656	0.1563	0.6771	2
C2_mixed_model_G125	ChIP_HepG2		mixed_effects	0.03575	0.1168	0.7595	4	fixed_effect_OLS_fallback	-0.07367	0.1971	0.7106	2
C2_mixed_model_G125	ChIP_either		mixed_effects	0.2237	0.1171	0.05609	4	fixed_effect_OLS_fallback	-0.1139	0.1979	0.5681	2
C2_mixed_model_G125	CollecTRI_87		mixed_effects	0.06788	0.1046	0.5164	4	mixed_effects	0.1843	0.1147	0.1083	3
C2_mixed_model_G125	ChIP_HepG2		mixed_effects	0.21	0.1147	0.06696	4	mixed_effects	0.2819	0.1233	0.02228	3
C2_mixed_model_G125	ChIP_either		mixed_effects	0.09172	0.1195	0.4428	4	mixed_effects	0.2345	0.1241	0.05886	3
C2_mixed_model_G125	CollecTRI_87		mixed_effects	-0.05678	0.09181	0.5363	4	fixed_effect_OLS_fallback	0.1578	0.09592	0.1071	2
C2_mixed_model_G125	ChIP_HepG2		mixed_effects	0.08022	0.1061	0.4496	4	fixed_effect_OLS_fallback	0.0863	0.1212	0.4801	2
C2_mixed_model_G125	ChIP_either		mixed_effects	-0.0002289	0.1128	0.9984	4	fixed_effect_OLS_fallback	0.1901	0.132	0.1568	2
C2_mixed_model_G125	CollecTRI_87		fixed_effect_OLS_fallback	0.06141	0.1385	0.6602	1	fixed_effect_OLS_fallback	-0.001811	0.1698	0.9916	1
C2_mixed_model_G125	ChIP_HepG2		fixed_effect_OLS_fallback	-0.09309	0.1812	0.6108	1	fixed_effect_OLS_fallback	-0.1825	0.2262	0.4284	1
C2_mixed_model_G125	ChIP_either		fixed_effect_OLS_fallback	0.008165	0.164	0.9606	1	fixed_effect_OLS_fallback	-0.05241	0.208	0.8034	1
C2_mixed_model_G125	CollecTRI_87		fixed_effect_OLS_fallback	0.116	0.1276	0.368	1	fixed_effect_OLS_fallback	0.1346	0.1771	0.4589	0
C2_mixed_model_G125	ChIP_HepG2		fixed_effect_OLS_fallback	0.2263	0.1486	0.1347	1	fixed_effect_OLS_fallback	0.1456	0.2378	0.5495	0
C2_mixed_model_G125	ChIP_either		fixed_effect_OLS_fallback	0.08615	0.1422	0.5476	1	fixed_effect_OLS_fallback	0.1526	0.2226	0.5035	0

Table S2. Merged-patient sensitivity analysis (GSE151530 same-patient multiregion samples merged; 32 samples from 25 patients): mixed-effects model comparison. Because merging same-patient samples can lift a subtype above the per-patient cell-count threshold, merged models can include more patients than the original models (e.g., Proliferating CD4: 5 versus 7 patients).

subtype	regulon	model_orig	n_patients_used_orig	beta_orig	se_orig	p_value_orig	model_merged	n_patients_used_merged	beta_merged	se_merged	p_value_merged	d_beta
CD4 NR4A+ early-activated	CollecTRI_87	mixed_effects	23	0.1202	0.0176	8.473e-12	mixed_effects	18	0.1079	0.0165	6.268e-11	-0.01237
CD4 NR4A+ early-activated	ChIP_HepG2	mixed_effects	23	0.1475	0.01964	5.891e-14	mixed_effects	18	0.1596	0.01825	2.221e-18	0.01211
CD4 NR4A+ early-activated	ChIP_either	mixed_effects	23	0.1172	0.01961	2.284e-09	mixed_effects	18	0.1234	0.01853	2.761e-11	0.006204
CD4 other	CollecTRI_87	mixed_effects	22	0.009642	0.02113	0.6481	mixed_effects	17	-0.006755	0.02043	0.7409	-0.0164
CD4 other	ChIP_HepG2	mixed_effects	22	-0.04602	0.02278	0.04338	mixed_effects	17	-0.04055	0.02174	0.06218	0.005471
CD4 other	ChIP_either	mixed_effects	22	0.03425	0.02342	0.1437	mixed_effects	17	0.0301	0.02269	0.1846	-0.004153
CD8 other	CollecTRI_87	mixed_effects	11	0.1321	0.05244	0.01177	mixed_effects	9	0.02422	0.04857	0.618	-0.1079
CD8 other	ChIP_HepG2	mixed_effects	11	0.04934	0.06524	0.4495	mixed_effects	9	-0.01594	0.05969	0.7894	-0.06529
CD8 other	ChIP_either	mixed_effects	11	0.1034	0.05931	0.08135	mixed_effects	9	0.01478	0.05463	0.7868	-0.08859
Cytotoxic CD8	CollecTRI_87	mixed_effects	11	0.1186	0.04754	0.01257	mixed_effects	9	0.1343	0.04451	0.002559	0.0156
Cytotoxic CD8	ChIP_HepG2	mixed_effects	11	0.1326	0.05071	0.008927	mixed_effects	9	0.1252	0.04713	0.007884	-0.007365
Cytotoxic CD8	ChIP_either	mixed_effects	11	0.08623	0.05181	0.09604	mixed_effects	9	0.1193	0.0489	0.01468	0.03311
Effector memory CD4	CollecTRI_87	mixed_effects	21	0.001415	0.01734	0.935	mixed_effects	16	0.02552	0.01682	0.1292	0.02411
Effector memory CD4	ChIP_HepG2	mixed_effects	21	-0.03163	0.0195	0.1048	mixed_effects	16	-0.02224	0.01858	0.2312	0.009394
Effector memory CD4	ChIP_either	mixed_effects	21	-0.003462	0.01996	0.8623	mixed_effects	16	0.04321	0.01964	0.02783	0.04667
Effector memory CD8	CollecTRI_87	mixed_effects	13	0.08912	0.02223	6.118e-05	mixed_effects	10	0.09223	0.02044	6.43e-06	0.003109
Effector memory CD8	ChIP_HepG2	mixed_effects	13	0.1221	0.02602	2.705e-06	mixed_effects	10	0.08029	0.02378	0.0007341	-0.04181
Effector memory CD8	ChIP_either	mixed_effects	13	0.1076	0.02477	1.398e-05	mixed_effects	10	0.1296	0.02297	1.695e-08	0.02199
Exhausted CD8	CollecTRI_87	mixed_effects	14	0.06686	0.02818	0.01766	mixed_effects	13	0.09501	0.02681	0.0003946	0.02815
Exhausted CD8	ChIP_HepG2	mixed_effects	14	0.05056	0.03158	0.1094	mixed_effects	13	0.05404	0.02981	0.06989	0.003482
Exhausted CD8	ChIP_either	mixed_effects	14	0.06693	0.0319	0.03589	mixed_effects	13	0.1418	0.031	4.78e-06	0.07486
Naive/CM CD4	CollecTRI_87	mixed_effects	13	0.03689	0.03434	0.2827	mixed_effects	11	0.03082	0.03233	0.3405	-0.006074
Naive/CM CD4	ChIP_HepG2	mixed_effects	13	-0.03687	0.03573	0.3021	mixed_effects	11	-0.01138	0.03372	0.7357	0.02549
Naive/CM CD4	ChIP_either	mixed_effects	13	0.02041	0.03854	0.5965	mixed_effects	11	0.01217	0.03677	0.7408	-0.008242
Proliferating CD4	CollecTRI_87	mixed_effects	5	-0.01147	0.119	0.9232	mixed_effects	7	-0.01242	0.09832	0.8995	-0.0009514
Proliferating CD4	ChIP_HepG2	mixed_effects	5	0.03737	0.1292	0.7724	mixed_effects	7	0.1867	0.1009	0.06424	0.1493
Proliferating CD4	ChIP_either	mixed_effects	5	0.1074	0.124	0.3861	mixed_effects	7	0.1059	0.1088	0.3305	-0.001546
Treg	CollecTRI_87	mixed_effects	11	0.1352	0.04433	0.00229	mixed_effects	10	0.1193	0.04285	0.00535	-0.01587
Treg	ChIP_HepG2	mixed_effects	11	0.113	0.04892	0.02084	mixed_effects	10	0.1011	0.04684	0.03091	-0.01196
Treg	ChIP_either	mixed_effects	11	0.1211	0.04992	0.01525	mixed_effects	10	0.09863	0.04846	0.04184	-0.02249

Table S3. Public T-cell long-read transcriptome screen: per-dataset CREM-locus classification counts, including datasets that could not be screened.

dataset	accession	cell_type	condition	platform	evidence_level	n_P1	n_P2_ICER	n_other_sense	n_antisense	evaluable_ge20	notes
TRAILS (Inamo et al. 2024)	DRA016285	15 T-cell subsets + PBMC	ex vivo resting	ONT cDNA	isoform-level	22	0	0	0	yes	22/22 CREM-locus isoforms initiate at P1; P1 detected in all 15 subsets and PBMC (8-61 per subset; Th17=52, CD8 effector=61, Fra2+ aTreg=43); 0 P2/ICER in the 210,019-isoform matrix; separate +68 kb downstream-promoter isoforms also present
Woolley et al. 2025	GSE229971 (GSM7182107-110)	CD4 T cells	activated 4 h/16 h/48 h/120 h	ONT	isoform-level	34	0	0	0	yes	34/34 collapsed isoforms initiate at P1 (5' ends chr10:35,126, 841-35,127,032); 0 P2 at all time points
Woolley et al. 2022	GSE202327 (GSM6108477/478)	CD4 and CD8 T cells	resting	ONT	isoform-level	3	0	0	0	no (n=3)	3/3 isoforms initiate at P1; below the >=20 quantitative-evaluability threshold, reported for completeness
E-MTAB-10797	ERP130787 (ERR6357 499/500/501)	T cells	per study of origin	ONT directRNA	read-level	4	1	7	5	no (n=17)	pooled across 3 replicates; 17 splice-filtered locus reads; below the >=20 threshold, reported for completeness
Fanari et al. 2025 (activated)	SRP520260 / SRR29847359	T cells	activated	ONT directRNA	read-level	48	0	1	0	partial	partial screen: ~423,347 reads aligned before transfer truncation; 50 primary locus alignments = 48 P1 (all spliced, sense-strand, mapq 60) + 1 unspliced P2-window read (608 nt, nascent, not counted as mature P2) + 1 other-sense; 0 antisense; one supplementary alignment not counted as an independent read
Fanari et al. 2025 (naive)	SRP520260 / SRR29847356	T cells	naive	ONT directRNA	read-level					no	not evaluable: file corruption during transfer left 17,656 mappable reads (2 locus reads)
Sato et al. 2025	doi:10.3390/ijms26136302	T cells	-	ONT	read-level					no	data available on request; not publicly screenable
Doyle et al. 2026	doi:10.3389/fgene.2026.1782221	T cells	-	ONT	read-level					no	CREM-locus coverage too sparse
SG-NEx HepG2 (context, previously reported)	SG-NEx directRNA rep6	HepG2 hepatoma	-	ONT directRNA	read-level	42	3	-	-	yes	previously reported (Figure 6); 93% P1, 95% Wilson CI [82%, 98%]

dataset	accession	cell_type	condition	platform	evidence_level	n_P1	n_P2_ICER	n_other_sense	n_antisense	evaluable_ge20	notes
TRAILS pilot (context, previously reported)	DRR465281	EM CD8 T cells	ex vivo	ONT	read-level	2	0	1	63	no (n=3)	previously reported (Table S8); caption corrected to 2 P1 + 1 other of 3 sense-classifiable reads

Table S4. Per-subtype dissociation of CREM mRNA from CREM-regulon activity: extended per-cohort statistics, bootstrap confidence intervals, and full mixed-effects model outputs.

Table S4 (part 1 of 3)

cohort	subtype	regulon	n_cells	n_patients	n_patients_ge10cells	n_CREM_pos	rho_full	p_full	rho_CREMpos	p_CREMpos	q_full_BH	q_CREMpos_BH	dissociation_index_full
GSE125449	CD4 NR4A+ early-activated	ChIP_HepG2	395	11	4	180	0.1363	0.0067	0.1904	0.0105	0.0222	0.0429	0.8637
GSE125449	CD4 other	ChIP_HepG2	223	11	7	95	0.1077	0.1087	0.2544	0.0129	0.2718	0.0429	0.8923
GSE125449	CD8 other	ChIP_HepG2	58	9	2	24	0.4198	0.001	0.3843	0.0637	0.0104	0.1592	0.5802
GSE125449	Cytotoxic CD8	ChIP_HepG2	38	10	1	14	0.216	0.1927	-0.1077	0.714	0.3211	0.8474	0.784
GSE125449	Effector memory CD4	ChIP_HepG2	236	12	6	158	0.179	0.0058	0.244	0.002	0.0222	0.0201	0.821
GSE125449	Effector memory CD8	ChIP_HepG2	168	11	4	81	-0.0172	0.8249	0.0217	0.8474	0.8249	0.8474	0.9828
GSE125449	Exhausted CD8	ChIP_HepG2	80	8	4	47	0.1646	0.1445	-0.1891	0.2031	0.289	0.4062	0.8354
GSE125449	Naive/CM CD4	ChIP_HepG2	134	11	4	84	0.0352	0.6868	-0.0438	0.6925	0.7631	0.8474	0.9648
GSE125449	Proliferating CD4	ChIP_HepG2	37	9	1	22	-0.0918	0.5891	0.0548	0.8087	0.7363	0.8474	0.9082
GSE125449	Treg	ChIP_HepG2	48	10	1	40	0.1592	0.2797	0.0756	0.6429	0.3995	0.8474	0.8408
GSE151530	CD4 NR4A+ early-activated	ChIP_HepG2	4046	29	23	2226	0.1884	0	0.0563	0.0079	0	0.0787	0.8116
GSE151530	CD4 other	ChIP_HepG2	2285	31	22	1186	-0.0147	0.4837	0.043	0.1387	0.5375	0.6934	0.9853
GSE151530	CD8 other	ChIP_HepG2	632	28	11	249	0.0413	0.2994	-0.0413	0.5163	0.4276	0.9802	0.9587
GSE151530	Cytotoxic CD8	ChIP_HepG2	617	27	11	339	0.0901	0.0252	0.0081	0.8822	0.0504	0.9802	0.9099
GSE151530	Effector memory CD4	ChIP_HepG2	3448	32	21	2299	0.0187	0.2724	0.005	0.8119	0.4276	0.9802	0.9813
GSE151530	Effector memory CD8	ChIP_HepG2	2792	29	13	1647	0.1408	0	0.0186	0.4503	0	0.9802	0.8592
GSE151530	Exhausted CD8	ChIP_HepG2	1408	28	14	861	0.0789	0.003	-0.01	0.769	0.0102	0.9802	0.9211
GSE151530	Naive/CM CD4	ChIP_HepG2	850	25	13	561	-0.0247	0.4725	-0.0002	0.9965	0.5375	0.9965	0.9753
GSE151530	Proliferating CD4	ChIP_HepG2	176	28	5	91	0.0023	0.9759	0.057	0.5918	0.9759	0.9802	0.9977
GSE151530	Treg	ChIP_HepG2	671	25	11	504	0.1057	0.0062	0.0356	0.4248	0.0154	0.9802	0.8943
GSE125449	CD4 NR4A+ early-activated	ChIP_either	395	11	4	180	0.0914	0.0697	0.1076	0.1506	0.1789	0.3011	0.9086
GSE125449	CD4 other	ChIP_either	223	11	7	95	0.0924	0.1689	0.2765	0.0067	0.2414	0.0334	0.9076
GSE125449	CD8 other	ChIP_either	58	9	2	24	0.4553	0.0003	0.4209	0.0406	0.0016	0.1352	0.5447
GSE125449	Cytotoxic CD8	ChIP_either	38	10	1	14	0.2416	0.1439	0.0505	0.8637	0.2399	0.8637	0.7584
GSE125449	Effector memory CD4	ChIP_either	236	12	6	158	0.2863	0	0.3608	0	0.0001	0	0.7137
GSE125449	Effector memory CD8	ChIP_either	168	11	4	81	0.1394	0.0716	0.057	0.6133	0.1789	0.7666	0.8606
GSE125449	Exhausted CD8	ChIP_either	80	8	4	47	0.0998	0.3784	-0.2605	0.077	0.473	0.1924	0.9002
GSE125449	Naive/CM CD4	ChIP_either	134	11	4	84	0.1312	0.1307	0.0936	0.3973	0.2399	0.6621	0.8688
GSE125449	Proliferating CD4	ChIP_either	37	9	1	22	0.0888	0.6011	0.0841	0.7097	0.6679	0.7886	0.9112
GSE125449	Treg	ChIP_either	48	10	1	40	0.0163	0.9126	-0.0857	0.5989	0.9126	0.7666	0.9837
GSE151530	CD4 NR4A+ early-activated	ChIP_either	4046	29	23	2226	0.204	0	0.0622	0.0033	0	0.0067	0.796
GSE151530	CD4 other	ChIP_either	2285	31	22	1186	0.0807	0.0001	0.0874	0.0026	0.0002	0.0065	0.9193
GSE151530	CD8 other	ChIP_either	632	28	11	249	0.1233	0.0019	-0.0331	0.603	0.0024	0.603	0.8767
GSE151530	Cytotoxic CD8	ChIP_either	617	27	11	339	0.1567	0.0001	0.068	0.212	0.0002	0.2651	0.8433
GSE151530	Effector memory CD4	ChIP_either	3448	32	21	2299	0.1239	0	0.0766	0.0002	0	0.0024	0.8761
GSE151530	Effector memory CD8	ChIP_either	2792	29	13	1647	0.2168	0	0.0855	0.0005	0	0.0026	0.7832
GSE151530	Exhausted CD8	ChIP_either	1408	28	14	861	0.2282	0	0.1051	0.002	0	0.0065	0.7718
GSE151530	Naive/CM CD4	ChIP_either	850	25	13	561	0.0697	0.0422	0.0622	0.141	0.0469	0.235	0.9303

cohort	subtype	regulon	n_cells	n_patients	n_patients_ge10cells	n_CREM_pos	rho_full	p_full	rho_CREMpos	p_CREMpos	q_full_BH	q_CREMpos_BH	dissociation_index_full
GSE151530	Proliferating CD4	ChIP_either	176	28	5	91	0.0258	0.7337	0.1109	0.2955	0.7337	0.3283	0.9742
GSE151530	Treg	ChIP_either	671	25	11	504	0.1396	0.0003	0.0561	0.2085	0.0004	0.2651	0.8604
GSE125449	CD4 NR4A+ early-activated	CollecTRI_87	395	11	4	180	0.0956	0.0576	0.0766	0.307	0.1629	0.4385	0.9044
GSE125449	CD4 other	CollecTRI_87	223	11	7	95	0.1169	0.0815	0.1776	0.085	0.1629	0.17	0.8831
GSE125449	CD8 other	CollecTRI_87	58	9	2	24	0.3845	0.0029	0.3774	0.0691	0.0144	0.17	0.6155
GSE125449	Cytotoxic CD8	CollecTRI_87	38	10	1	14	0.2059	0.2148	0.3363	0.2398	0.3581	0.3997	0.7941
GSE125449	Effector memory CD4	CollecTRI_87	236	12	6	158	0.2159	0.0008	0.2747	0.0005	0.0084	0.0048	0.7841
GSE125449	Effector memory CD8	CollecTRI_87	168	11	4	81	0.1407	0.0688	0.0378	0.7373	0.1629	0.7373	0.8593
GSE125449	Exhausted CD8	CollecTRI_87	80	8	4	47	0.0629	0.5796	-0.2769	0.0595	0.644	0.17	0.9371
GSE125449	Naive/CM CD4	CollecTRI_87	134	11	4	84	0.0943	0.2783	0.196	0.074	0.3976	0.17	0.9057
GSE125449	Proliferating CD4	CollecTRI_87	37	9	1	22	0.1462	0.3878	0.0841	0.7097	0.4847	0.7373	0.8538
GSE125449	Treg	CollecTRI_87	48	10	1	40	0.0663	0.6543	-0.1135	0.4856	0.6543	0.607	0.9337
GSE151530	CD4 NR4A+ early-activated	CollecTRI_87	4046	29	23	2226	0.1834	0	0.0362	0.088	0	0.2199	0.8166
GSE151530	CD4 other	CollecTRI_87	2285	31	22	1186	0.0445	0.0334	0.0307	0.2911	0.0417	0.3639	0.9555
GSE151530	CD8 other	CollecTRI_87	632	28	11	249	0.1163	0.0034	-0.0469	0.4615	0.0049	0.5128	0.8837
GSE151530	Cytotoxic CD8	CollecTRI_87	617	27	11	339	0.1492	0.0002	0.0667	0.2205	0.0004	0.3639	0.8508
GSE151530	Effector memory CD4	CollecTRI_87	3448	32	21	2299	0.0908	0	0.0545	0.0089	0	0.0891	0.9092
GSE151530	Effector memory CD8	CollecTRI_87	2792	29	13	1647	0.1824	0	0.0576	0.0194	0	0.097	0.8176
GSE151530	Exhausted CD8	CollecTRI_87	1408	28	14	861	0.1668	0	0.048	0.1597	0	0.3194	0.8332
GSE151530	Naive/CM CD4	CollecTRI_87	850	25	13	561	0.062	0.071	0.0908	0.0316	0.0789	0.1053	0.938
GSE151530	Proliferating CD4	CollecTRI_87	176	28	5	91	-0.0401	0.5969	-0.1169	0.2696	0.5969	0.3639	0.9599
GSE151530	Treg	CollecTRI_87	671	25	11	504	0.1296	0.0008	0.0241	0.5891	0.0013	0.5891	0.8704

Table S4 (part 2 of 3)

cohort	subtype	regulon	dissociation_index_CREMpos	boot_rho_mean	boot_ci_lo	boot_ci_hi	n_boot_kept	model	beta	se	p_value	n_cells_used	n_patients_used
GSE125449	CD4 NR4A+ early-activated	ChIP_HepG2	0.8096	0.1368	0.0451	0.2292	1000	mixed_effects	0.1376	0.0601	0.0219	360	4
GSE125449	CD4 other	ChIP_HepG2	0.7456	0.106	-0.0181	0.2296	1000	mixed_effects	-0.0464	0.0681	0.4958	203	7
GSE125449	CD8 other	ChIP_HepG2	0.6157	0.4171	0.2136	0.609	1000	fixed_effect_OLS_fallback	0.4912	0.1363	0.0007	58	2
GSE125449	Cytotoxic CD8	ChIP_HepG2	0.8923	0.2086	-0.0742	0.4503	1000	fixed_effect_OLS_fallback	0.248	0.162	0.1346	38	1
GSE125449	Effector memory CD4	ChIP_HepG2	0.756	0.1789	0.0522	0.302	1000	mixed_effects	0.0286	0.0649	0.6589	220	6
GSE125449	Effector memory CD8	ChIP_HepG2	0.9783	-0.019	-0.1701	0.1297	1000	mixed_effects	0.0358	0.1168	0.7595	140	4
GSE125449	Exhausted CD8	ChIP_HepG2	0.8109	0.1611	-0.0206	0.3483	1000	mixed_effects	0.21	0.1147	0.067	69	4
GSE125449	Naive/CM CD4	ChIP_HepG2	0.9562	0.034	-0.1271	0.1955	1000	mixed_effects	0.0802	0.1061	0.4496	106	4
GSE125449	Proliferating CD4	ChIP_HepG2	0.9452	-0.092	-0.4017	0.2212	1000	fixed_effect_OLS_fallback	-0.0931	0.1812	0.6108	37	1
GSE125449	Treg	ChIP_HepG2	0.9244	0.1654	-0.1315	0.4338	1000	fixed_effect_OLS_fallback	0.2263	0.1486	0.1347	48	1
GSE151530	CD4 NR4A+ early-activated	ChIP_HepG2	0.9437	0.1891	0.1607	0.2165	1000	mixed_effects	0.1475	0.0196	0	4019	23
GSE151530	CD4 other	ChIP_HepG2	0.957	-0.0152	-0.055	0.0241	1000	mixed_effects	-0.046	0.0228	0.0434	2246	22
GSE151530	CD8 other	ChIP_HepG2	0.9587	0.0434	-0.0299	0.1159	1000	mixed_effects	0.0493	0.0652	0.4495	571	11
GSE151530	Cytotoxic CD8	ChIP_HepG2	0.9919	0.0898	0.0125	0.1675	1000	mixed_effects	0.1326	0.0507	0.0089	567	11

cohort	subtype	regulon	dissociation_index_CREMpos	boot_rho_mean	boot_ci_lo	boot_ci_hi	n_boot_kept	model	beta	se	p_value	n_cells_used	n_patients_used
GSE151530	Effector memory CD4	ChIP_HepG2	0.995	0.019	-0.0109	0.0513	1000	mixed_effects	-0.0316	0.0195	0.1048	3407	21
GSE151530	Effector memory CD8	ChIP_HepG2	0.9814	0.1414	0.1062	0.1762	1000	mixed_effects	0.1221	0.026	0	2721	13
GSE151530	Exhausted CD8	ChIP_HepG2	0.99	0.078	0.0262	0.1284	1000	mixed_effects	0.0506	0.0316	0.1094	1373	14
GSE151530	Naive/CM CD4	ChIP_HepG2	0.9998	-0.0235	-0.0906	0.0419	1000	mixed_effects	-0.0369	0.0357	0.3021	810	13
GSE151530	Proliferating CD4	ChIP_HepG2	0.943	0.0021	-0.1363	0.1374	1000	mixed_effects	0.0374	0.1292	0.7724	91	5
GSE151530	Treg	ChIP_HepG2	0.9644	0.1043	0.0289	0.1788	1000	mixed_effects	0.113	0.0489	0.0208	617	11
GSE125449	CD4 NR4A+ early-activated	ChIP_either	0.8924	0.0925	0.0017	0.1797	1000	mixed_effects	0.0863	0.0638	0.1761	360	4
GSE125449	CD4 other	ChIP_either	0.7235	0.0946	-0.0262	0.2131	1000	mixed_effects	-0.0357	0.0781	0.6481	203	7
GSE125449	CD8 other	ChIP_either	0.5791	0.4529	0.269	0.642	1000	fixed_effect_OLS_fallback	0.6133	0.165	0.0005	58	2
GSE125449	Cytotoxic CD8	ChIP_either	0.9495	0.2393	0.0443	0.4181	1000	fixed_effect_OLS_fallback	0.3266	0.2162	0.1396	38	1
GSE125449	Effector memory CD4	ChIP_either	0.6392	0.2844	0.1775	0.3991	1000	mixed_effects	0.0401	0.0755	0.5952	220	6
GSE125449	Effector memory CD8	ChIP_either	0.943	0.1363	-0.0022	0.2755	1000	mixed_effects	0.2237	0.1171	0.0561	140	4
GSE125449	Exhausted CD8	ChIP_either	0.7395	0.1015	-0.111	0.3004	1000	mixed_effects	0.0917	0.1195	0.4428	69	4
GSE125449	Naive/CM CD4	ChIP_either	0.9064	0.1313	-0.0182	0.2825	1000	mixed_effects	-0.0002	0.1128	0.9984	106	4
GSE125449	Proliferating CD4	ChIP_either	0.9159	0.0726	-0.254	0.3688	1000	fixed_effect_OLS_fallback	0.0082	0.164	0.9606	37	1
GSE125449	Treg	ChIP_either	0.9143	0.0243	-0.2754	0.3042	1000	fixed_effect_OLS_fallback	0.0861	0.1422	0.5476	48	1
GSE151530	CD4 NR4A+ early-activated	ChIP_either	0.9378	0.2042	0.1762	0.2314	1000	mixed_effects	0.1172	0.0196	0	4019	23
GSE151530	CD4 other	ChIP_either	0.9126	0.0793	0.0399	0.1172	1000	mixed_effects	0.0342	0.0234	0.1437	2246	22
GSE151530	CD8 other	ChIP_either	0.9669	0.1221	0.0552	0.189	1000	mixed_effects	0.1034	0.0593	0.0814	571	11
GSE151530	Cytotoxic CD8	ChIP_either	0.932	0.1563	0.0843	0.2277	1000	mixed_effects	0.0862	0.0518	0.096	567	11
GSE151530	Effector memory CD4	ChIP_either	0.9234	0.1239	0.0909	0.1567	1000	mixed_effects	-0.0035	0.02	0.8623	3407	21
GSE151530	Effector memory CD8	ChIP_either	0.9145	0.2178	0.1846	0.2516	1000	mixed_effects	0.1076	0.0248	0	2721	13
GSE151530	Exhausted CD8	ChIP_either	0.8949	0.228	0.1819	0.2755	1000	mixed_effects	0.0669	0.0319	0.0359	1373	14
GSE151530	Naive/CM CD4	ChIP_either	0.9378	0.0701	0.0061	0.1354	1000	mixed_effects	0.0204	0.0385	0.5965	810	13
GSE151530	Proliferating CD4	ChIP_either	0.8891	0.0232	-0.1023	0.1481	1000	mixed_effects	0.1074	0.124	0.3861	91	5
GSE151530	Treg	ChIP_either	0.9439	0.1391	0.0641	0.2102	1000	mixed_effects	0.1211	0.0499	0.0153	617	11
GSE125449	CD4 NR4A+ early-activated	CollecTRI_87	0.9234	0.0974	0.0054	0.1884	1000	mixed_effects	0.0952	0.0577	0.0989	360	4
GSE125449	CD4 other	CollecTRI_87	0.8224	0.1185	-0.0074	0.2348	1000	mixed_effects	0.0416	0.0693	0.5487	203	7
GSE125449	CD8 other	CollecTRI_87	0.6226	0.3808	0.1745	0.5728	1000	fixed_effect_OLS_fallback	0.4057	0.1401	0.0054	58	2
GSE125449	Cytotoxic CD8	CollecTRI_87	0.6637	0.2026	-0.0145	0.4037	1000	fixed_effect_OLS_fallback	0.2452	0.1812	0.1843	38	1
GSE125449	Effector memory CD4	CollecTRI_87	0.7253	0.2129	0.0748	0.3291	1000	mixed_effects	0.0398	0.0692	0.5656	220	6
GSE125449	Effector memory CD8	CollecTRI_87	0.9622	0.1434	0.0028	0.2773	1000	mixed_effects	0.222	0.1057	0.0357	140	4
GSE125449	Exhausted CD8	CollecTRI_87	0.7231	0.0648	-0.1406	0.2601	1000	mixed_effects	0.0679	0.1046	0.5164	69	4
GSE125449	Naive/CM CD4	CollecTRI_87	0.804	0.0947	-0.0814	0.2565	1000	mixed_effects	-0.0568	0.0918	0.5363	106	4
GSE125449	Proliferating CD4	CollecTRI_87	0.9159	0.1413	-0.1865	0.426	1000	fixed_effect_OLS_fallback	0.0614	0.1385	0.6602	37	1
GSE125449	Treg	CollecTRI_87	0.8865	0.0754	-0.244	0.3661	1000	fixed_effect_OLS_fallback	0.116	0.1276	0.368	48	1

cohort	subtype	regulon	dissociation_index_CREMpos	boot_rho_mean	boot_ci_lo	boot_ci_hi	n_boot_kept	model	beta	se	p_value	n_cells_used	n_patients_used
GSE151530	CD4 NR4A+ early-activated	CollecTRI_87	0.9638	0.1838	0.1548	0.2106	1000	mixed_effects	0.1202	0.0176	0	4019	23
GSE151530	CD4 other	CollecTRI_87	0.9693	0.0446	0.0034	0.0878	1000	mixed_effects	0.0096	0.0211	0.6481	2246	22
GSE151530	CD8 other	CollecTRI_87	0.9531	0.1158	0.0428	0.1938	1000	mixed_effects	0.1321	0.0524	0.0118	571	11
GSE151530	Cytotoxic CD8	CollecTRI_87	0.9333	0.147	0.0744	0.2192	1000	mixed_effects	0.1186	0.0475	0.0126	567	11
GSE151530	Effector memory CD4	CollecTRI_87	0.9455	0.0909	0.0577	0.1228	1000	mixed_effects	0.0014	0.0173	0.935	3407	21
GSE151530	Effector memory CD8	CollecTRI_87	0.9424	0.183	0.1481	0.2176	1000	mixed_effects	0.0891	0.0222	0.0001	2721	13
GSE151530	Exhausted CD8	CollecTRI_87	0.952	0.167	0.122	0.2137	1000	mixed_effects	0.0669	0.0282	0.0177	1373	14
GSE151530	Naive/CM CD4	CollecTRI_87	0.9092	0.0599	-0.0037	0.1252	1000	mixed_effects	0.0369	0.0343	0.2827	810	13
GSE151530	Proliferating CD4	CollecTRI_87	0.8831	-0.0368	-0.1611	0.0857	1000	mixed_effects	-0.0115	0.119	0.9232	91	5
GSE151530	Treg	CollecTRI_87	0.9759	0.1316	0.0601	0.2065	1000	mixed_effects	0.1352	0.0443	0.0023	617	11

Table S4 (part 3 of 3)

cohort	subtype	regulon	converged	rho_meta	ci_lo	ci_hi	p_meta	q_meta_BH	Q	I2_pct	dissociation_index
GSE125449	CD4 NR4A+ early-activated	ChIP_HepG2	True	0.1838	0.1552	0.2121	0	0	1.022	2.201	0.8162
GSE125449	CD4 other	ChIP_HepG2	True	-0.0039	-0.043	0.0353	0.8468	0.8494	3.025	66.94	0.9961
GSE125449	CD8 other	ChIP_HepG2	True	0.0739	-0.0009	0.1479	0.0529	0.0881	8.34	88.01	0.9261
GSE125449	Cytotoxic CD8	ChIP_HepG2	True	0.097	0.0204	0.1725	0.0132	0.0263	0.5522	0	0.903
GSE125449	Effector memory CD4	ChIP_HepG2	True	0.029	-0.0033	0.0612	0.0789	0.1127	5.742	82.58	0.971
GSE125449	Effector memory CD8	ChIP_HepG2	True	0.1321	0.0965	0.1673	0	0	3.935	74.58	0.8679
GSE125449	Exhausted CD8	ChIP_HepG2	True	0.0834	0.0327	0.1337	0.0013	0.0043	0.5533	0	0.9166
GSE125449	Naive/CM CD4	ChIP_HepG2	True	-0.0167	-0.0792	0.046	0.6023	0.7528	0.4064	0	0.9833
GSE125449	Proliferating CD4	ChIP_HepG2	True	-0.0132	-0.1483	0.1224	0.8494	0.8494	0.2528	0	0.9868
GSE125449	Treg	ChIP_HepG2	True	0.1091	0.0361	0.1809	0.0035	0.0086	0.1254	0	0.8909
GSE151530	CD4 NR4A+ early-activated	ChIP_HepG2	True	0.1838	0.1552	0.2121	0	0	1.022	2.201	0.8162
GSE151530	CD4 other	ChIP_HepG2	True	-0.0039	-0.043	0.0353	0.8468	0.8494	3.025	66.94	0.9961
GSE151530	CD8 other	ChIP_HepG2	True	0.0739	-0.0009	0.1479	0.0529	0.0881	8.34	88.01	0.9261
GSE151530	Cytotoxic CD8	ChIP_HepG2	True	0.097	0.0204	0.1725	0.0132	0.0263	0.5522	0	0.903
GSE151530	Effector memory CD4	ChIP_HepG2	True	0.029	-0.0033	0.0612	0.0789	0.1127	5.742	82.58	0.971
GSE151530	Effector memory CD8	ChIP_HepG2	True	0.1321	0.0965	0.1673	0	0	3.935	74.58	0.8679
GSE151530	Exhausted CD8	ChIP_HepG2	True	0.0834	0.0327	0.1337	0.0013	0.0043	0.5533	0	0.9166
GSE151530	Naive/CM CD4	ChIP_HepG2	True	-0.0167	-0.0792	0.046	0.6023	0.7528	0.4064	0	0.9833
GSE151530	Proliferating CD4	ChIP_HepG2	True	-0.0132	-0.1483	0.1224	0.8494	0.8494	0.2528	0	0.9868
GSE151530	Treg	ChIP_HepG2	True	0.1091	0.0361	0.1809	0.0035	0.0086	0.1254	0	0.8909
GSE125449	CD4 NR4A+ early-activated	ChIP_either	True	0.1942	0.1658	0.2224	0	0	4.751	78.95	0.8058
GSE125449	CD4 other	ChIP_either	True	0.0817	0.0427	0.1205	0	0.0001	0.0283	0	0.9183
GSE125449	CD8 other	ChIP_either	True	0.1523	0.0784	0.2245	0.0001	0.0001	6.829	85.36	0.8477
GSE125449	Cytotoxic CD8	ChIP_either	True	0.1613	0.0856	0.2352	0	0.0001	0.2594	0	0.8387
GSE125449	Effector memory CD4	ChIP_either	True	0.1345	0.1027	0.1661	0	0	6.304	84.14	0.8655
GSE125449	Effector memory CD8	ChIP_either	True	0.2125	0.1779	0.2467	0	0	0.9969	0	0.7875
GSE125449	Exhausted CD8	ChIP_either	True	0.2217	0.1727	0.2695	0	0	1.274	21.51	0.7783
GSE125449	Naive/CM CD4	ChIP_either	True	0.078	0.0155	0.1399	0.0145	0.0161	0.4387	0	0.922
GSE125449	Proliferating CD4	ChIP_either	True	0.0362	-0.0997	0.1708	0.6023	0.6023	0.1136	0	0.9638
GSE125449	Treg	ChIP_either	True	0.1319	0.0592	0.2032	0.0004	0.0005	0.6505	0	0.8681
GSE151530	CD4 NR4A+ early-activated	ChIP_either	True	0.1942	0.1658	0.2224	0	0	4.751	78.95	0.8058

cohort	subtype	regulon	converged	rho_meta	ci_lo	ci_hi	p_meta	q_meta_BH	Q	I2_pct	dissociation_index
GSE151530	CD4 other	ChIP_either	True	0.0817	0.0427	0.1205	0	0.0001	0.0283	0	0.9183
GSE151530	CD8 other	ChIP_either	True	0.1523	0.0784	0.2245	0.0001	0.0001	6.829	85.36	0.8477
GSE151530	Cytotoxic CD8	ChIP_either	True	0.1613	0.0856	0.2352	0	0.0001	0.2594	0	0.8387
GSE151530	Effector memory CD4	ChIP_either	True	0.1345	0.1027	0.1661	0	0	6.304	84.14	0.8655
GSE151530	Effector memory CD8	ChIP_either	True	0.2125	0.1779	0.2467	0	0	0.9969	0	0.7875
GSE151530	Exhausted CD8	ChIP_either	True	0.2217	0.1727	0.2695	0	0	1.274	21.51	0.7783
GSE151530	Naive/CM CD4	ChIP_either	True	0.078	0.0155	0.1399	0.0145	0.0161	0.4387	0	0.922
GSE151530	Proliferating CD4	ChIP_either	True	0.0362	-0.0997	0.1708	0.6023	0.6023	0.1136	0	0.9638
GSE151530	Treg	ChIP_either	True	0.1319	0.0592	0.2032	0.0004	0.0005	0.6505	0	0.8681
GSE125449	CD4 NR4A+ early-activated	CollecTRI_87	True	0.1757	0.147	0.2041	0	0	2.866	65.1	0.8243
GSE125449	CD4 other	CollecTRI_87	True	0.0509	0.0118	0.0899	0.0108	0.0135	1.067	6.283	0.9491
GSE125449	CD8 other	CollecTRI_87	True	0.1391	0.065	0.2117	0.0002	0.0004	4.21	76.25	0.8609
GSE125449	Cytotoxic CD8	CollecTRI_87	True	0.1523	0.0764	0.2264	0.0001	0.0002	0.1138	0	0.8477
GSE125449	Effector memory CD4	CollecTRI_87	True	0.0988	0.0667	0.1307	0	0	3.594	72.17	0.9012
GSE125449	Effector memory CD8	CollecTRI_87	True	0.1801	0.1449	0.2147	0	0	0.2848	0	0.8199
GSE125449	Exhausted CD8	CollecTRI_87	True	0.1615	0.1115	0.2106	0	0	0.8115	0	0.8385
GSE125449	Naive/CM CD4	CollecTRI_87	True	0.0663	0.0037	0.1284	0.0379	0.0421	0.1204	0	0.9337
GSE125449	Proliferating CD4	CollecTRI_87	True	-0.0094	-0.1446	0.1262	0.8928	0.8928	0.9984	0	0.9906
GSE125449	Treg	CollecTRI_87	True	0.1256	0.0528	0.1971	0.0007	0.0011	0.1722	0	0.8744
GSE151530	CD4 NR4A+ early-activated	CollecTRI_87	True	0.1757	0.147	0.2041	0	0	2.866	65.1	0.8243
GSE151530	CD4 other	CollecTRI_87	True	0.0509	0.0118	0.0899	0.0108	0.0135	1.067	6.283	0.9491
GSE151530	CD8 other	CollecTRI_87	True	0.1391	0.065	0.2117	0.0002	0.0004	4.21	76.25	0.8609
GSE151530	Cytotoxic CD8	CollecTRI_87	True	0.1523	0.0764	0.2264	0.0001	0.0002	0.1138	0	0.8477
GSE151530	Effector memory CD4	CollecTRI_87	True	0.0988	0.0667	0.1307	0	0	3.594	72.17	0.9012
GSE151530	Effector memory CD8	CollecTRI_87	True	0.1801	0.1449	0.2147	0	0	0.2848	0	0.8199
GSE151530	Exhausted CD8	CollecTRI_87	True	0.1615	0.1115	0.2106	0	0	0.8115	0	0.8385
GSE151530	Naive/CM CD4	CollecTRI_87	True	0.0663	0.0037	0.1284	0.0379	0.0421	0.1204	0	0.9337
GSE151530	Proliferating CD4	CollecTRI_87	True	-0.0094	-0.1446	0.1262	0.8928	0.8928	0.9984	0	0.9906
GSE151530	Treg	CollecTRI_87	True	0.1256	0.0528	0.1971	0.0007	0.0011	0.1722	0	0.8744

Table S5. ChIP-anchored CREM target gene lists (ChIP_HepG2-27 and ChIP_either-42 regulon variants). The table contains 88 rows: the 87 CollecTRI-derived targets plus CREM itself (weight = 1.0), which is retained as the regulon seed.

target	weight	in_var	expr_pct_pos	mean_expr	has_hepg2	has_gm	max_signal_hepg2	max_signal_gm	closest_dist_hepg2	closest_dist_gm
LIPE	1	True	0.013	0.017	False	True	0	18.53		3586
FSHB	-1	False	0	0	False	False	0	0		
INS	1	False	0	0	False	False	0	0		
TAF4	1	True	0.018	0.021	True	False	70.18	0	1141	
IL2	1	True	0.016	0.025	False	False	0	0		
APRT	1	True	0.591	1.024	False	False	0	0		
TH	-1	False	0	0	True	False	67.94	0	4388	
GAST	1	False	0	0	False	False	0	0		
AANAT	1	False	0	0	True	True	82.2	51.33	32	84
CALM1	1	True	0.822	1.904	False	True	0	39.73		328
PCK1	1	True	0.007	0.008	True	False	367	0	88	
AGTRAP	1	True	0.137	0.194	False	False	0	0		
AGTR1	1	True	0.003	0.004	False	False	0	0		
FSHR	1	False	0	0	False	False	0	0		
FASLG	-1	True	0.091	0.126	False	False	0	0		
CREBBP	1	True	0.107	0.138	False	True	0	40.49		110
CREB1	-1	True	0.113	0.15	True	True	603.5	194.5	142	174
VDR	1	True	0.011	0.013	False	False	0	0		
PRKACA	1	True	0.029	0.035	True	True	333.3	65.65	37	4
BCL2	-1	True	0.193	0.273	False	False	0	0		
PSMA4	1	True	0.328	0.498	False	True	0	18.62		78
LHB	1	False	0	0	False	True	0	35.21		595
CELA2A	1	False	0	0	False	False	0	0		
ETV3	1	True	0.102	0.131	True	True	115.9	75.33	159	1
CCNA2	-1	True	0.016	0.019	True	True	810.6	211.7	119	57
GPX4	1	True	0.529	0.877	True	True	204.5	30.23	58	484
CCK	1	True	0	0	False	False	0	0		
FOS	-1	True	0.589	1.393	True	True	535.2	117.7	47	81
STAR	1	False	0	0	False	False	0	0		
CRH	1	False	0	0	True	False	57.12	0	21	
PTH	1	False	0	0	False	False	0	0		
CD247	-1	True	0.345	0.518	False	False	0	0		
CCL4	1	True	0.441	1.23	True	True	112	28.18	1	67
CISH	1	True	0.067	0.1	False	False	0	0		
CREB3L4	1	True	0.007	0.009	True	False	451.4	0	3724	
CCND1	-1	True	0.025	0.032	False	False	0	0		
CHGA	1	False	0	0	False	False	0	0		
TNF	-1	True	0.113	0.189	False	False	0	0		
MC2R	1	False	0	0	False	False	0	0		
PGK2	1	False	0	0	False	False	0	0		
GJD2	1	False	0	0	False	False	0	0		
ADRA1D	1	False	0	0	True	False	61.28	0	3968	
SLC5A5	1	False	0	0	False	False	0	0		
BRAF	1	True	0.053	0.064	True	True	2224	167.8	26	42
BGLAP	1	True	0.002	0.003	False	False	0	0		
CYP51A1	-1	True	0.012	0.012	True	True	167.8	61.71	3	64
HMGCR	1	True	0.036	0.043	True	True	516.9	84.47	577	697

target	weight	in_var	expr_pct_pos	mean_expr	has_hepg2	has_gm	max_signal_hepg2	max_signal_gm	closest_dist_hepg2	closest_dist_gm
CD86	1	True	0.004	0.005	False	True	0	32.62		145
CRABP2	-1	True	0.002	0.002	False	False	0	0		
PER1	1	True	0.098	0.127	True	True	278.2	74.37	78	34
NFATC1	1	True	0.058	0.079	False	True	0	18.52		3215
SYK	1	True	0.006	0.007	False	False	0	0		
IL17A	1	True	0.008	0.017	False	False	0	0		
ABCC8	1	False	0	0	False	False	0	0		
PTGER2	1	True	0.161	0.234	False	False	0	0		
MYD88	1	True	0.061	0.076	True	False	58.45	0	1018	
NOTCH1	1	True	0.036	0.045	False	True	0	39.12		82
CD8B	1	True	0.24	0.437	False	False	0	0		
CD8A	1	True	0.307	0.6	False	False	0	0		
CAMK4	1	True	0.147	0.199	True	False	51.4	0	2147	
TSHR	1	False	0	0	False	False	0	0		
PTPRC	1	True	0.78	1.788	False	True	0	32.36		401
ADRB1	-1	True	0.006	0.007	False	True	0	18.63		7
ACE	1	True	0.007	0.01	True	False	83.05	0	891	
PRL	1	False	0	0	False	False	0	0		
NGF	-1	True	0	0.001	False	False	0	0		
TESK1	1	True	0.031	0.038	True	True	193.5	30.4	987	981
IFNG	1	True	0.225	0.476	False	True	0	22.39		4095
IL5RA	1	True	0.002	0.003	True	True	2865	57.58	41	152
ACR	1	True	0	0	False	False	0	0		
ACOT7	1	True	0.043	0.052	False	False	0	0		
CCNA1	1	False	0	0	False	False	0	0		
DUSP1	-1	True	0.689	1.468	True	True	1125	52.61	105	55
DUSP4	-1	True	0.238	0.418	False	True	0	30.79		54
G6PD	-1	True	0.082	0.108	False	True	0	19.23		48
LDHC	1	True	0.002	0.003	False	False	0	0		
PLAT	1	True	0.016	0.022	False	False	0	0		
TBP	1	True	0.024	0.029	True	True	960.1	197.7	228	139
ZAP70	1	True	0.199	0.281	False	False	0	0		
JUN	-1	True	0.506	1.12	True	True	164	34.35	27	90
CSF2	1	False	0	0	False	False	0	0		
IL4	1	False	0	0	False	False	0	0		
CYP19A1	-1	False	0	0	False	False	0	0		
PDE3A	-1	True	0	0	False	False	0	0		
GSTP1	-1	True	0.358	0.568	False	True	0	16.33		90
NR3C1	1	True	0.304	0.467	False	True	0	67.11		159
THOP1	1	True	0.03	0.037	True	True	538.4	161.5	1835	1987
CREM	1	True	0.584	1.289	True	True	265.9	139.1	92	114

Table S6. SCENIC de-novo regulon inference: per-seed CREM regulon targets, bZIP-family recovery across seeds, cross-subtype comparison statistics, and the 29-gene bZIP/CREB pathway whitelist used to supplement SCENIC inference (four sections).

**Per-seed CREM regulon targets**				
seed	n_motifs_for_CREM	n_targets	targets	
42	1	8	BCL2L11, CNOT6L, CXCR4, FAM214A, FOSL2, MAF, ZNF331, ZNF596	
123	0	0	(CREM not recovered)	
2024	0	0	(CREM not recovered)	
intersection	-	0	(empty: CREM not in seeds 123 and 2024)	
union	-	8	BCL2L11, CNOT6L, CXCR4, FAM214A, FOSL2, MAF, ZNF331, ZNF596	
bZIP family recovery per seed				
TF	42	123	2024	n_seeds
ATF1	1	1	1	3
BATF3	1	1	1	3
FOSL2	1	1	1	3
BATF	1	1	1	3
FOSL1	1	1	1	3
JUND	1	1	1	3
FOS	1	1	1	3
JUN	1	1	1	3
ATF4	0	1	1	2
ATF3	1	0	1	2
JUNB	1	1	0	2
FOSB	0	1	1	2
ATF5	0	1	1	2
ATF2	0	0	1	1
CREB3	0	0	1	1
CREB1	0	1	0	1
ATF7	1	0	0	1
CREM	1	0	0	1
Cross-subtype meta-rho comparison				
subtype	ChIP_HepG2	ChIP_either	CollecTRI_87	SCENIC_seed42
CD4 NR4A+ early-activated	0.1838	0.1942	0.1757	0.2159
CD4 other	-0.0039	0.0817	0.0509	0.2884
CD8 other	0.0739	0.1523	0.1391	0.3798
Cytotoxic CD8	0.097	0.1613	0.1523	0.2464
Effector memory CD4	0.029	0.1345	0.0988	0.1539
Effector memory CD8	0.1321	0.2125	0.1801	0.164
Exhausted CD8	0.0834	0.2217	0.1615	0.132
Naive/CM CD4	-0.0167	0.078	0.0663	0.2509
Proliferating CD4	-0.0132	0.0362	-0.0094	0.07916
Treg	0.1091	0.1319	0.1256	0.09843
29-gene bZIP/CREB pathway whitelist (SCENIC supplementation)				
gene				
CREM				
CREB1				
CREB3				

Per-seed CREM regulon targets				
CREB3L1				
CREB3L2				
ATF1				
ATF2				
ATF3				
ATF4				
ATF5				
ATF6				
ATF6B				
ATF7				
JUN				
JUNB				
JUND				
FOS				
FOSB				
FOSL1				
FOSL2				
BATF				
BATF2				
BATF3				
MAF				
MAFB				
MAFF				
MAFG				
MAFK				
NFE2L2				

Table S7. TCR clonal expansion analysis (GSE98638): full per-subtype statistics and mixed-effects model outputs (two sections).

**Per-subtype clone-size associations**							
subtype	n_cells	metric	spearman_rho	pval	qval_BH		
Exhausted_CD8	286	CREM__CollecTRI_87_score	0.08449	0.1541	0.2774		
Exhausted_CD8	286	CREM__ChIP_HepG2_score	0.05217	0.3794	0.6761		
Exhausted_CD8	286	CREM__ChIP_either_score	0.008123	0.8912	0.8912		
Exhausted_CD8	286	CREM_log1p	0.1213	0.04043	0.2507		
MAIT_CD8	88	CREM__CollecTRI_87_score	-0.2688	0.01133	0.05098		
MAIT_CD8	88	CREM__ChIP_HepG2_score	-0.07414	0.4924	0.6761		
MAIT_CD8	88	CREM__ChIP_either_score	-0.1586	0.1401	0.4162		
MAIT_CD8	88	CREM_log1p	-0.1151	0.2855	0.367		
EffectorMem_CD8	236	CREM__CollecTRI_87_score	-0.06186	0.3441	0.4424		
EffectorMem_CD8	236	CREM__ChIP_HepG2_score	0.03208	0.6239	0.7019		
EffectorMem_CD8	236	CREM__ChIP_either_score	0.009876	0.88	0.8912		
EffectorMem_CD8	236	CREM_log1p	0.05042	0.4408	0.4959		
EffectorMem_CD4	331	CREM__CollecTRI_87_score	0.1814	0.0009157	0.008242		
EffectorMem_CD4	331	CREM__ChIP_HepG2_score	0.2542	2.795e-06	2.515e-05		
EffectorMem_CD4	331	CREM__ChIP_either_score	0.2275	2.928e-05	0.0002635		
EffectorMem_CD4	331	CREM_log1p	-0.08876	0.107	0.2507		
Effector_CD8	52	CREM__CollecTRI_87_score	0.2186	0.1194	0.2688		
Effector_CD8	52	CREM__ChIP_HepG2_score	0.2214	0.1147	0.3442		
Effector_CD8	52	CREM__ChIP_either_score	0.06802	0.6318	0.8291		
Effector_CD8	52	CREM_log1p	-0.2446	0.0805	0.2507		
NKlike_CD4	64	CREM__CollecTRI_87_score	0.2326	0.06433	0.193		
NKlike_CD4	64	CREM__ChIP_HepG2_score	0.3184	0.01034	0.04655		
NKlike_CD4	64	CREM__ChIP_either_score	0.2737	0.02862	0.1288		
NKlike_CD4	64	CREM_log1p	-0.1548	0.222	0.367		
Tfh	120	CREM__CollecTRI_87_score	-0.07006	0.447	0.5029		
Tfh	120	CREM__ChIP_HepG2_score	0.02447	0.7908	0.7908		
Tfh	120	CREM__ChIP_either_score	0.04722	0.6085	0.8291		
Tfh	120	CREM_log1p	-0.1461	0.1114	0.2507		
Naive_CD4	73	CREM__CollecTRI_87_score	-0.1463	0.2169	0.3254		
Naive_CD4	73	CREM__ChIP_HepG2_score	-0.07544	0.5259	0.6761		
Naive_CD4	73	CREM__ChIP_either_score	-0.1569	0.185	0.4162		
Naive_CD4	73	CREM_log1p	-0.03847	0.7466	0.7466		
CTLA4_CD4	363	CREM__CollecTRI_87_score	0.001398	0.9788	0.9788		
CTLA4_CD4	363	CREM__ChIP_HepG2_score	0.05699	0.2788	0.6273		
CTLA4_CD4	363	CREM__ChIP_either_score	0.02428	0.6448	0.8291		
CTLA4_CD4	363	CREM_log1p	0.05918	0.2607	0.367		
Mixed-effects model outputs							
subtype	n_cells	n_patients	metric	beta	se	pval	qval_BH
Exhausted_CD8	286	5	CollecTRI_87	0.0668	0.1303	0.6082	0.782
Exhausted_CD8	286	5	ChIP_HepG2	-0.2207	0.1477	0.1351	0.447
Exhausted_CD8	286	5	ChIP_either	-0.1184	0.1561	0.448	0.7638
Exhausted_CD8	286	5	CREM_mRNA	0.04491	0.1291	0.7279	0.9878
MAIT_CD8	88	5	CollecTRI_87	-0.587	0.2518	0.01974	0.1777
MAIT_CD8	88	5	ChIP_HepG2	-0.3227	0.3072	0.2936	0.5041
MAIT_CD8	88	5	ChIP_either	-0.3853	0.2924	0.1875	0.7638
MAIT_CD8	88	5	CREM_mRNA	-0.002726	0.1784	0.9878	0.9878

**Per-subtype clone-size associations**							
EffectorMem_CD8	236	5	CollecTRI_87	-0.2398	0.1568	0.1262	0.5218
EffectorMem_CD8	236	5	ChIP_HepG2	-0.01588	0.175	0.9277	0.991
EffectorMem_CD8	236	5	ChIP_either	-0.05112	0.1785	0.7745	0.7888
EffectorMem_CD8	236	5	CREM_mRNA	0.0204	0.1366	0.8813	0.9878
EffectorMem_CD4	331	5	CollecTRI_87	0.1694	0.1804	0.3479	0.5218
EffectorMem_CD4	331	5	ChIP_HepG2	0.4699	0.2104	0.0255	0.2295
EffectorMem_CD4	331	5	ChIP_either	0.3452	0.1929	0.07348	0.6613
EffectorMem_CD4	331	5	CREM_mRNA	0.09529	0.1511	0.5282	0.9878
Effector_CD8	52	5	CollecTRI_87	0.3003	0.2818	0.2867	0.5218
Effector_CD8	52	5	ChIP_HepG2	0.003451	0.3058	0.991	0.991
Effector_CD8	52	5	ChIP_either	-0.08521	0.3182	0.7888	0.7888
Effector_CD8	52	5	CREM_mRNA	0.04548	0.1546	0.7686	0.9878
NKlike_CD4	64	5	CollecTRI_87	0.3038	0.2832	0.2834	0.5218
NKlike_CD4	64	5	ChIP_HepG2	0.4045	0.3147	0.1986	0.447
NKlike_CD4	64	5	ChIP_either	0.3157	0.3326	0.3425	0.7638
NKlike_CD4	64	5	CREM_mRNA	-0.2278	0.1597	0.1538	0.7607
Tfh	120	5	CollecTRI_87	0.02581	0.329	0.9375	0.9375
Tfh	120	5	ChIP_HepG2	0.4058	0.4219	0.3361	0.5041
Tfh	120	5	ChIP_either	0.2752	0.41	0.5021	0.7638
Tfh	120	5	CREM_mRNA	-0.5298	0.3852	0.1691	0.7607
Naive_CD4	73	5	CollecTRI_87	-0.8927	0.8594	0.2989	0.5218
Naive_CD4	73	5	ChIP_HepG2	0.1933	0.9864	0.8447	0.991
Naive_CD4	73	5	ChIP_either	-0.6981	1.058	0.5092	0.7638
Naive_CD4	73	5	CREM_mRNA	0.3252	0.6844	0.6346	0.9878
CTLA4_CD4	363	4	CollecTRI_87	-0.0604	0.1553	0.6973	0.7844
CTLA4_CD4	363	4	ChIP_HepG2	0.2248	0.1724	0.1923	0.447
CTLA4_CD4	363	4	ChIP_either	0.05769	0.1753	0.7421	0.7888
CTLA4_CD4	363	4	CREM_mRNA	0.04446	0.163	0.785	0.9878

Table S8. T-cell long-read pilot (TRAILS DRR465281): CREM-locus read classification counts (66 post-filter reads: 2 P1, 0 P2, 1 other-sense, and 63 antisense; i.e., 2 P1 and 1 other-sense among the 3 sense-classifiable reads).

classification	n_reads
P1 (CREMτ)	2
P2 (ICER)	0
other_sense	1
antisense	63

Table S9. Anti-PD-1 bulk cohort analysis (GSE202069): full per-patient statistics.

metric	n_R	n_NR	R_mean	NR_mean	Cohen_d	AUC	wilcox_U	wilcox_p	wilcox_q_BH
CollecTRI_87	8	8	0.007835	-0.005122	0.03162	0.5	32	1	1
ChIP_HepG2	8	8	0.7722	0.8179	-0.2146	0.4062	26	0.5737	1
ChIP_either	8	8	1.255	1.391	-0.3406	0.4844	31	0.9591	1
CREM_log2TPM	8	8	2.482	2.309	1.029	0.7188	46	0.1605	0.6421
